# Long noncoding RNA LINC00239 inhibits ferroptosis in colorectal cancer by binding to Keap1 to stabilize Nrf2

**DOI:** 10.1038/s41419-022-05192-y

**Published:** 2022-08-29

**Authors:** Yuying Han, Xiaoliang Gao, Nan Wu, Yirong Jin, He Zhou, Weijie Wang, Hao Liu, Yi Chu, Jiayi Cao, Mingzuo Jiang, Suzhen Yang, Yanting Shi, Xin Xie, Fulin Chen, Ying Han, Wen Qin, Bing Xu, Jie Liang

**Affiliations:** 1 Key Laboratory of Resource Biology and Biotechnology in Western China, Ministry of Education. School of Medicine, Northwest University, 229 Taibai North Road, 710069 Xi’an, China; 2 State Key Laboratory of Cancer Biology, National Clinical Research Center for Digestive Diseases and Xijing Hospital of Digestive Diseases, Air Force Military Medical University, 710032 Xi’an, China; 3 Department of Gastroenterology, the Affiliated Drum Tower Hospital of Nanjing University Medical School, 210002 Nanjing, Jiangsu China; 4 Department of Gastroenterology and Hepatology, Jinling Hospital, Medical School of Nanjing University, Nanjing, China; 5 Department of Gastroenterology, Second Affiliated Hospital of Xi’an Jiaotong University, Xi’an, China; 6 State Key Laboratory of Military Stomatology, National Clinical Research Center for Oral Diseases, Shaanxi Clinical Research Center for Oral Diseases, Xi’an, China

**Keywords:** Colorectal cancer, Cell death

## Abstract

Ferroptosis, a novel regulated cell death induced by iron-dependent lipid peroxidation, plays an important role in tumor development and drug resistance. Long noncoding RNAs (lncRNAs) are associated with various types of cancer. However, the precise roles of many lncRNAs in tumorigenesis remain elusive. Here we explored the transcriptomic profiles of lncRNAs in primary CRC tissues and corresponding paired adjacent non-tumor tissues by RNA-seq and found that LINC00239 was significantly overexpressed in colorectal cancer tissues. Abnormally high expression of LINC00239 predicts poorer survival and prognosis in colorectal cancer patients. Concurrently, we elucidated the role of LINC00239 as a tumor-promoting factor in CRC through in vitro functional studies and in vivo tumor xenograft models. Importantly, overexpression of LINC00239 decreased the anti-tumor activity of erastin and RSL3 by inhibiting ferroptosis. Collectively, these data suggest that LINC00239 plays a novel and indispensable role in ferroptosis by nucleotides 1–315 of LINC00239 to interact with the Kelch domain (Nrf2-binding site) of Keap1, inhibiting Nrf2 ubiquitination and increasing Nrf2 protein stability. Considering the recurrence and chemoresistance constitute the leading cause of death in colorectal cancer (CRC), ferroptosis induction may be a promising therapeutic strategy for CRC patients with low LINC00239 expression.

## Introduction

Despite tremendous improvements in detection and treatment, colorectal cancer (CRC) remains one of the most aggressive malignancies of the digestive system, with the third highest incidence and second highest mortality worldwide [1, 2]. Uncontrollable cell proliferation and continuous inhibition of cell death are common causes of poor prognosis in patients with CRC [3, 4]. Unfortunately, the molecular and genetic alterations in these processes remain elusive.

Cell death is strictly regulated by complex intracellular and extracellular signals, which are essential for various biological processes (including redox homeostasis imbalance, development, and disease) [5]. Ferroptosis is a new form of regulated cell death that involves the accumulation of iron-dependent lipid peroxides (lipid-ROS) and causes fatal cell damage [6]. Central to controlling redox homeostasis in carcinogenesis, NF-E2-related factor 2 (Nrf2) has attracted much attention due to its important role in mediating adaptation to oncogene-stimulated oxidative stress [7]. Nuclear translocation and constitutive activation of Nrf2 protect cancer cells from death and induce cell proliferation [8]. In particular, ferroptosis, a new type of regulated cell death (RCD) in the presence of iron-driven lipid peroxidation, has been implicated in Nrf2-mediated carcinogenesis [9, 10]. Mounting evidence indicates that ferroptosis exerts an antitumor effect on tumor progression [11–13], yet the biological and mechanistic details underlying this complex process remain unclear.

Long noncoding RNAs (lncRNAs) are a class of transcripts that lack protein-coding capacity and have lengths greater than 200 nucleotides [14, 15]. Tens of thousands of lncRNAs may be encoded in the human genome, but the precise roles of a large number of them remain elusive [16]. Recent studies have shown that lncRNAs are powerful and multifunctional cell regulators during tumorigenesis and development [17]. LncRNAs play important roles in the occurrence, metastasis, and drug resistance of colorectal cancer [18]. Depending on their subcellular localization, lncRNAs function in various forms [19]. Nuclear lncRNAs are involved in transcriptional regulation in cis and trans, regulation of chromosomal interactions, transcription factor trapping, chromatin circularization, gene methylation, transcription factor recruitment, and chromatin modification [20–22]. Cytoplasmic lncRNAs can regulate target protein levels by interacting with proteins, mRNAs, or micro-RNAs [23]. Accumulating evidence has revealed that lncRNAs are important regulators of oxidative stress during the development of tumors [24–26]. However, the more specific roles of lncRNAs in CRC under ferroptosis remain largely unknown.

In this study, we confirmed that LINC00239 is a ferroptosis suppressor in CRC. LINC00239 promotes CRC proliferation by interacting with Kelch-like ECH-associated protein 1 (Keap1), causing instability of the Keap1/Nrf2 complex. Therefore, LINC00239 enhanced Nrf2 protein stability by suppressing its ubiquitination and promoted CRC development. Importantly, Nrf2 also promotes LINC00239 transcription in a positive feedback manner. LINC00239 inhibition in combination with ferroptosis induction might be a promising therapeutic strategy for CRC patients.

## Materials and methods

### Patients and follow-up

The study protocol was approved by the ethics committee of Air Force Military Medical University (Shaanxi, China). Written informed consent was obtained from all participants in this study. Cohort I included freshly sampled CRC tissues with healthy adjacent tissues collected between January 2005 and December 2007 from 174 adult patients who underwent surgery at Xijing Hospital of the Fourth Military Medical University (Xi’an, China). Cohort II included CRC tissue samples from 180 adult CRC patients at the Shanghai Outdo Biotech Co., Ltd (Shanghai, China). All patients were staged pathologically based on the American Joint Committee on Cancer (AJCC)/International Union against Cancer criteria. All the research was carried out in accordance with the provisions of the Declaration of Helsinki of 1975. None of the patients had received radiotherapy or chemotherapy prior to surgery.

### Cell culture and treatments

Human CRC cell lines RKO, HCT116, CaCo2, SW480, SW620, and normal intestinal epithelial cells (FHC) were purchased from American Type Culture Collection (ATCC, USA). All cells were cultured in Dulbecco’s modified Eagle’s medium (DMEM, Gibco, Carlsbad, CA, USA) supplemented with 10% fetal calf serum, penicillin, and streptomycin (Gibco, Carlsbad, CA, USA) at 37 °C in an atmosphere containing 5% CO_2_. The cell lines were tested for mycoplasma contamination before use to ensure that they were mycoplasma-free. All drugs were ordered from MedChemExpress unless otherwise indicated. All drug use was performed according to the manufacturer’s instructions. All small interfering RNAs (siRNAs) were purchased from TsingKe Technology (Beijing, China). Lipofectamine 2000 (Thermo Fisher Scientific, USA) was used to transfect siRNA (50 nM) into colorectal cells, while nonspecific siRNA (50 nM) was used as a negative control. The sequences of siRNAs are listed in Supplementary Table S1.

### RNA isolation and qRT-PCR

For RNA-seq and qRT-PCR analysis, a MiniBEST Universal RNA Extraction Kit (TaKaRa, Japan) was used to isolate RNA, and a one-step PrimeScript RT-PCR kit was used to reverse transcribe 1 µg of total RNA (TaKaRa, Japan). TB Green^®^ Fast qPCR Mix (TaKaRa, Japan) was used for quantitative PCR with three repeated reactions with the primers listed in Supplementary Table S2. Using the ddCt method to compare with the 18 S level, the relative RNA expression level was calculated and normalized with respect to the control sample [27].

### Plasmids and cloning

Gibson cloning was used for all vectors. For gene knockdown, we cloned the sgRNA in Supplementary Table S1 into the pLentiRNACRISPR-hU6-DR-RfxCas13 (Addgene no. 138147) vector [28]. For gene overexpression analysis, the human LINC00239, Nrf2, and Keap1 full-length open-reading frames were subcloned into plenti-CMV-Luc-Puro (Addgene no. 17477) [29]. All constructs were confirmed by Sanger sequencing. All primers used for molecular cloning and primer sequences are shown in Supplementary Table S2.

### In vivo experiments

To clarify the role of LINC00239 in vivo, we used 4-week-old male BALB/c nude mice provided by the Experimental Animal Center of the Air Force Military Medical University. HCT116 or SW620 cells (1 × 10^7^ cells) were injected subcutaneously into the right flanks of these mice to establish a CRC xenograft model. One week after the injection of cells, the volume of xenografts was continuously monitored (once a week). Four weeks later, the xenografts were removed, and the weights were measured. All experimental procedures were approved by the Animal Care and Use Committee of Shanghai Air Force Military Medical University.

### Immunoblotting

The cells were collected by scraping and lysed for 15 min on ice in lysis buffer containing protease and phosphatase inhibitors. The cell lysate was centrifuged at 12,000 rpm and 4 °C for 15 min. SDS loading buffer was added to the supernatant, and then the sample was heated at 95 °C for 5 min before loading on the polyacrylamide gel. Western blotting was performed as previously described. The antibodies are listed in Supplementary Table S3.

### RNA FISH

Single-molecule RNA FISH was performed as previously described [27]. The probe was designed by the online probe designer at https://www.biosearchtech.com/products/rna-fish/ and labeled with Cy3. The probe for LINC00239 is listed in Supplementary Table S1.

### Immunofluorescence staining

For Keap1 and Nrf2 immunofluorescence staining assays, cells were fixed with 2% PFA at room temperature for 15 min. Then, the cells were permeated with 0.5% Triton X-100 for 15 min on ice and washed three times with PBS. The cells were then subjected to a blocking step and incubated with anti-Keap1 or anti-Nrf2 antibody at 4 °C overnight, followed by incubation with a fluorescent secondary antibody. The nuclei were counterstained with DAPI, and images were obtained by a laser confocal microscope [30]. The antibodies are listed in Supplementary Table S3.

### Co-immunoprecipitation (co-IP)

Co-IP was performed as described before [31]. In short, the input and immunoprecipitation samples were analyzed by western blotting using various antibodies at the specified dilution: Keap1 antibody, Nrf2 antibody, and normal rabbit IgG. The antibodies are listed in Supplementary Table S3.

### Chromatin immunoprecipitation

The procedure was performed as previously described [32]. In short, the cells were cross-linked with 1% formaldehyde for 10 min at room temperature and neutralized by adding glycine to a final concentration of 0.125 M for 5 min. After washing twice with cold PBS, the cells were collected and suspended in cold lysis buffer (10 mM Tris-Cl, pH 8.0, 85 mM KCl, 0.5% NP40, 5 mM EDTA, 0.25% Triton, and protease inhibitor). After 15 min of incubation on ice, the nuclei were harvested, resuspended in cold lysis buffer, and sonicated to obtain 200–500 base pair DNA fragments. Magnetic beads coated with specific antibody or IgG control were added to the lysis buffer and incubated overnight. The next day, the beads were washed 7 times with washing buffer (50 mM HEPES, pH 7.5, 500 mM LiCl, 1 mM EDTA, 1% NP-40, and 0.7% sodium deoxycholate), then washed once with TE buffer, after which the protein–DNA complex was eluted. After reverse cross-linking at 55 °C overnight, DNA was extracted and analyzed by qRT-PCR. All primers are listed in Supplementary Table S2.

### Immunohistochemistry (IHC)

In the immunohistochemical analysis of our own cohort, samples were independently assessed by two pathologists blinded to the clinical characteristics of the patients on the basis of staining intensity and region of protein expression in the samples. The percentage of positive cells was scored as 0 (<10%), 1 (10–40%), 2 (40–70%), or 3 (>70%), and the immunostaining intensity was scored as 0 (no staining), 1 (weak staining), 2 (moderate staining), or 3 (strong staining). The final immune response score was calculated as staining intensity score × percentage of positive cells. A score of 0-6 indicates that the expression of the target is considered “LINC00239 (−)”, while a score of 7–9 is considered “LINC00239 ( + )”.

### Data analyses and statistics

Relative RNA levels were normalized to 18 S RNA levels. All statistical analyses were performed using the GraphPad Prism 8.0 software package and SPSS 22.0 statistical software package (Abbott Laboratories, USA) for Windows. Data are presented as the means ± SD of at least three independent experiments. ^ns^*P* > 0.05, *P < 0.05, **P < 0.01, ***P < 0.001, ****P < 0.0001, Student’s *t* test.

### Reporting summary

Further information on experimental design is available in the Nature Research Reporting Summary linked to this paper.

## Results

### Overexpression of LINC00239 promotes CRC proliferation and indicates a worse prognosis

**Fig. 1:**
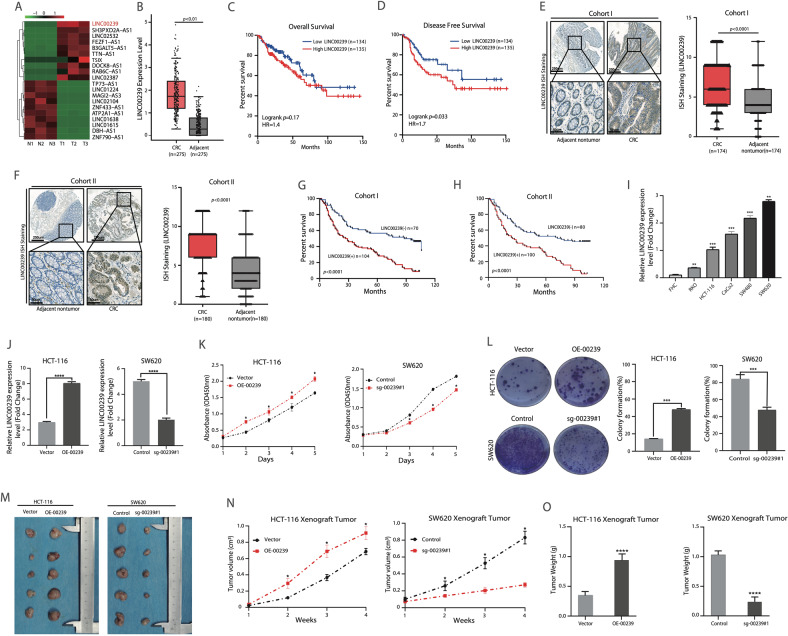
Overexpression of LINC00239 promotes CRC metastasis and indicates a worse prognosis. **A** Heatmap of K-means clustering of differentially expressed lncRNAs (log_2_FC > 5.0, *P*adj. < 0.05) during three paired CRC (T1–T3) and adjacent normal (N1–N3) tissues. LINC00239 is marked in red. **B** Statistical analysis of LINC00239 expression in TCGA of colorectal cancer and normal tissues, paired *t* test. Red indicates colorectal cancer tissue (*n* = 275), and gray indicates adjacent tissue (*n* = 275). **C** Kaplan–Meier overall survival (OS) analysis of LINC00239 status in CRC patients (TCGA, *n* = 269). **D** Kaplan–Meier disease-free survival (DFS) analysis of LINC00239 status in CRC patients (TCGA, *n* = 269). **E**, **F** The representative image of ISH staining of LINC00239 in CRC and adjacent nontumor tissues microarray. The scale bars represent 250 μm (low magnification) and 50 μm (high magnification). Right: The ISH score of LINC00239 in CRC and adjacent nontumor samples in Cohort I (*n* = 174) and Cohort II (*n* = 180). **G**, **H** Kaplan–Meier analysis of the relationships of LINC00239 expression and overall survival times or the recurrence rates in two independent CRC cohorts. **I** Real-time PCR analyses of the expression of LINC00239 in CRC cell lines and normal intestinal epithelial cells (FHC). **J** Real-time PCR analysis of LINC00239 expression in HCT-116 and SW620 cells after lentivirus transfection. **K** Cell viability in the indicated CRC cell lines by CCK-8 assay. **L** Cell viability in the indicated CRC cell lines by colony-formation assay. **M**–**O** Nude mice are shown after injection of HCT-116 and SW620 cells stably expressing the control vector, LINC00239 or Ko-LINC00239 expression plasmids. Tumor formation was monitored at the images (**M**), indicated times (**N**), and weights (**O**) were recorded (*n* = 5). Data shown represent mean ± SD from three independent experiments. ns *P* > 0.05, *P < 0.05, **P < 0.01, ***P < 0.001, ****P < 0.0001, Student’s *t* test.

To investigate the role of lncRNAs in colorectal cancer, we first examined lncRNA expression profiles in three colorectal cancer tissue samples and adjacent normal tissues using the RNA-seq. 71 lncRNAs were upregulated, and 215 were downregulated by more than five-fold (*P*adj. < 0.05)), including LINC00239 (Fig. 1A and Supplementary Table S4). Using 5′- and 3′-rapid amplification of the cDNA ends, LINC00239 was found to be 652-nucleotide (nt) long (Supplementary Fig. S1A, B). The gene is identical to the sequence LINC00239 in the UCSC database. To further address the clinical significance of LINC00239, analysis of the public database (GEPIA) [33] showed upregulation of LINC00239 in CRC tissues (Fig. 1B), and this upregulation predicted poor Overall survival (OS) (Fig. 1C). At the same time, we also found that the Disease-free survival (DFS) show a significant difference (Fig. 1D). Consistent with public database, we examined the LINC00239 expression of CRC and adjacent nontumor specimens in two human CRC cohorts by ISH staining. The LINC00239 levels were markedly upregulated in CRC compared with that in adjacent nontumor tissues (Fig. 1E, F). The elevated LINC00239 expression was associated with worse tumor size and higher AJCC stage (Table 1). CRC patients with positive LINC00239 expression had shorter overall survival than CRC patients with negative expression of LINC00239 (Fig. 1G, H). To investigate the biological function of LINC00239 in CRC, we first used qRT-PCR to analyze the expression of LINC00239 in different CRC cells and normal intestinal epithelial cells (FHC). The results showed that LINC00239 expression was upregulated in all CRC cell lines compared with the FHC cell line (Fig. 1I). Then, HCT-116 cells with relatively low LINC00239 expression and SW620 cells with relatively high LINC00239 expression were selected to construct HCT-116-OE-00239 and SW620-sg-00239#1 stable cell lines (Fig. 1J and Supplementary Fig. S1C). Upregulation of LINC00239 expression elevated the HCT-116 cells’ proliferation abilities. Downregulation of LINC00239 expression suppressed the SW620 cells’ proliferation capabilities (Fig. 1G, L). Similarly, an in vivo tumorigenesis experiment also showed a larger tumor size and heavier tumor weight in mice injected with LINC00239-overexpressing HCT-116-OE-00239 cells. In contrast, a smaller tumor size and lighter tumor weight were observed with SW620-sg-00239#1 cells, further confirming the oncogenic role of LINC00239 in CRC (Fig. 1M–O).

**Table 1:** Correlation between LINC00239 expression and clinicopathological characteristics of CRCs in two independent cohorts of human CRC tissues.

Clinicopathological variables	Cohort I (*n* = 174)		*P* value	Cohort II (*n* = 180)		*P* value
	Tumor LINC00239 expression			Tumor LINC00239 expression		
	Negative (*n* = 70)	Positive (*n* = 104)		Negative (*n* = 80)	Positive (*n* = 100)	
Age (years)						
>60	38	58	0.846996	47	58	0.919218
≤60	32	46		33	42	
Sex						
Female	40	56	0.668081	35	45	0.866815
Male	30	48		45	55	
Tumor location						
Right colon	20	36	0.659117	20	30	0.755572
Left colon	27	39		33	39	
Rectum	23	29		27	31	
Tumor size						
<5 cm	62	12	6.8684E-24	69	7	1.0527E-26
≥5 cm	8	92		11	93	
Tumor invasion						
T1	26	9	7.6288E-10	29	3	1.2432E-13
T2	28	21		31	17	
T3	12	30		15	37	
T4	4	44		5	43	
Lymph node metastasis						
Absent	40	54	0.498114	42	58	0.460574
Present	30	50		38	42	
AJCC stage						
Stage I	30	5	1.1328E-18	27	4	1.4835E-12
Stage II	28	8		31	14	
Stage III	9	54		16	45	
Stage IV	3	37		6	37	

### LINC00239 upregulates colorectal cancer cell growth by inhibiting ferroptosis

**Fig. 2:**
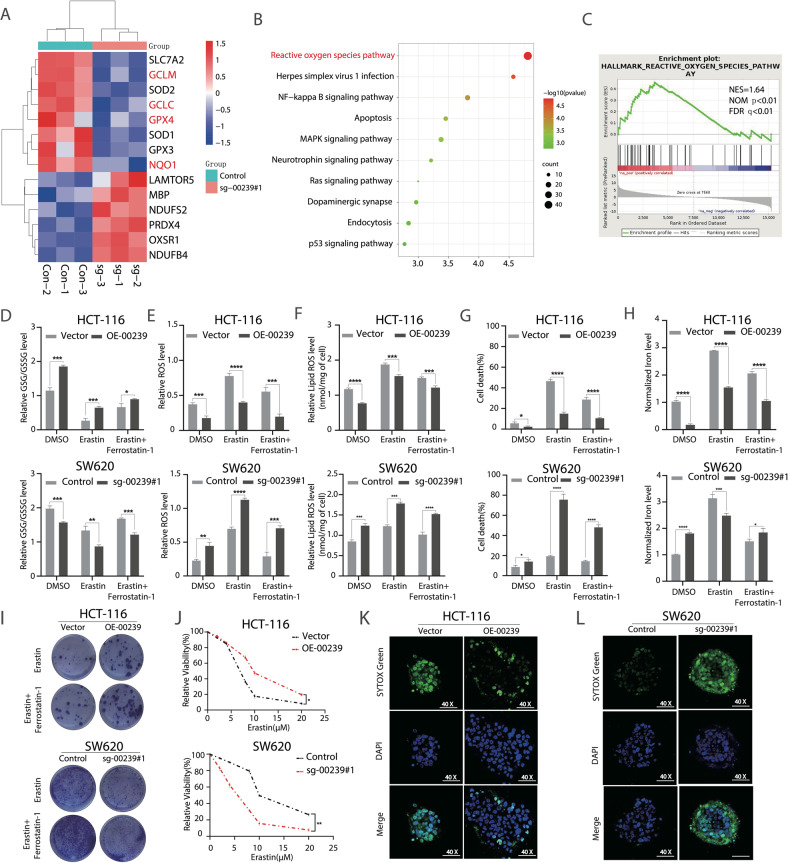
LINC00239 upregulates colorectal cancer cell proliferation by inhibiting ferroptosis. **A** RNA-seq-based heatmap indicating the changes of ferroptosis-related genes in SW620 cells knockout LINC00239. **B** KEGG analysis of LINC00239-related gene co-expression networks from the sequencing data. **C** GSEA analysis of LINC00239-related gene co-expression networks from the sequencing data. **D**–**H** GSH/GSSG ratios (**D**), ROS levels (**E**), Lipid ROS (**F**), cell viability (**G**), and Fe^2+^ concentration (**H**) was measured in the four indicated cell lines after treated with 10 μM erastin for 48 h and the addition of 2 µM ferrostatin-1 (Fer-1). **I**, **J** Colony-formation and CCK-8 assay to evaluate the cell viability of LINC00239 on colorectal cancer cells after treated with 2 μM erastin and the addition of 2 µM ferrostatin-1 (Fer-1). **K**, **L** Spheroids generated from the indicated cell lines were cultured for 96 h and treated with 15 μM erastin for 48 h. Dead cells were stained by SYTOX Green (original magnification, ×40). Data shown represent mean ± SD from three independent experiments. ns *P* > 0.05, *P < 0.05, **P < 0.01, ***P < 0.001, ****P < 0.0001, Student’s *t* test.

To determine the molecular mechanism underlying LINC00239-mediated CRC proliferation, RNA-seq were used to analyze the gene expression changes in LINC00239-knockout CRC cell lines. The results showed that a large number of genes mediating oxidative stress were significantly downregulated in SW620 cells with LINC00239 knockout (Fig. 2A–C). Ferroptosis involves the accumulation of iron-dependent lipid peroxides (lipid-ROS) and leads to lethal cellular damage [34]. The process of ferroptosis is divided into upstream (ROS production) and downstream (execution of ferroptosis) [35]. We induced ferroptosis using erastin in HCT-116-OE-00239 and SW620-sg-00239#1. Because GSG/GSSG, ROS, lipid ROS, and Fe^2+^ are essential for ferroptosis process, we further measured their concentrations in erastin- and ferrostatin-1-treated CRC cells. We found that knockout of LINC00239 sensitized cells to ferroptosis, as evidenced by reduced GSH/GSSG levels, accompanied by enhanced ROS accumulation, lipid ROS, accumulation of Fe^2+^ and an increased percentage of cell death upon erastin induction (Fig. 2D–H). The ferroptosis inhibitor ferrostatin-1 partly reversed erastin-induced ferroptosis in SW620 LINC00239-knockout cells (Fig. 2D–H). Conversely, ectopic expression of LINC00239 showed resistance to ferroptosis upon treatment with erastin plus ferrostatin-1 (Fig. 2D–H). Moreover, overexpression of LINC00239 enhanced clonogenicity and cell viability upon ferroptosis induction. Conversely, knockout of LINC00239 impaired both the clonogenicity and cell viability induced by ferroptosis induction (Fig. 2I, J). 3D tumor spheroid experiments further validated the inhibitory ferroptotic effect of LINC00239, as indicated by lower numbers of dead cells in LINC00239-overexpressing HCT116 cells and higher numbers of dead cells in LINC00239-knockout SW620 cells stained with SYTOX Green (Fig. 2K, L). Taken together, these results indicate that LINC00239 acts as an oncogenic lncRNA in CRC.

### LINC00239 interacts with Kelch-like ECH-associated protein 1

**Fig. 3:**
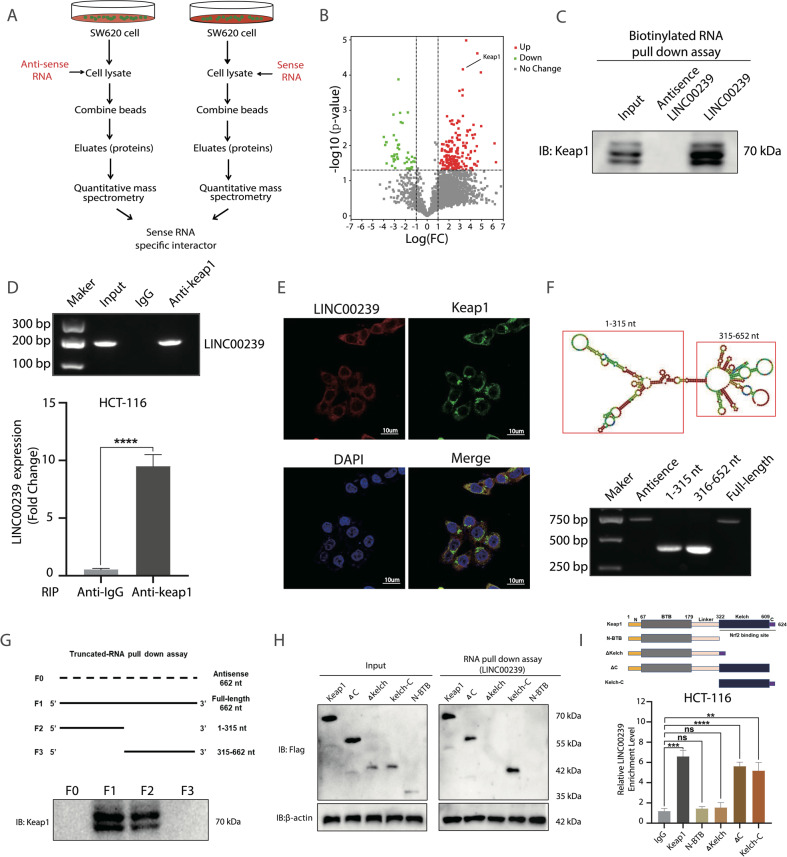
LINC00239 interacts with Keap1. **A** Proteomic approach to identify the LINC00239-specific interactors. **B** Proteome-wide accurate quantification and significance. **C** Western blot of the proteins from anti-sense LINC00239 and LINC00239 pull-down assays. **D** RNA immunoprecipitation experiments were performed using anti-Keap1 antibody, and specific primers were used to detect LINC00239. **E** ImmunoFISH images of LINC00239 and Keap1 in SW620 cells. **F** Top, the predicted secondary structure of LINC00239. Bottom, the in vitro-transcribed full-length LINC00239 and deletion fragments with the correct sizes indicated. **G** Deletion mapping of the Keap1-binding domain in LINC00239. Top, diagrams of full-length LINC00239 and the deletion fragments. Bottom, immunoblot analysis for Keap1 in the protein samples pulled down by different LINC00239 constructs. **H** The RNA Pull-down analysis of FLAG-tagged Keap1 versus domain truncation mutants) retrieved by in vitro-transcribed biotinylated LINC00239. **I** RIP assays show the association of the Kelch domain with LINC00239. Data shown represent mean ± SD from three independent experiments. ns *P* > 0.05, **P < 0.01, ***P < 0.001, ****P < 0.0001, Student’s *t* test.

Because lncRNAs may function through their interactions with other proteins, we hypothesized that LINC00239 might interact with certain cellular proteins to regulate biological functions. To explore the mechanism of LINC00239 in ferroptosis regulation and CRC proliferation, we first used RNA pulldown assays (Fig. 3A) and mass spectrometry analysis, we identified a LINC00239-protein complex in cell lysates generated from SW620 cells, antisense of LINC00239 served as a negative control in these experiments. Among the many proteins identified Kelch Like ECH Associated Protein 1(Keap1) were the most interesting (Fig. 3B and supplementary Table S5). We validated the presence of Keap1 in an intact complex from independent RNA pulldown assays in SW620 cells (Fig. 3C). Moreover, RNA immunoprecipitation (RIP) assays confirmed an enrichment of LINC00239 in the complexes precipitated with the antibody against Keap1 in HCT-116 cell (Fig. 3D). In addition, FISH staining also revealed colocalization between LINC00239 and Keap1 (Fig. 3E). To further clarify the molecular mechanism of the interaction between LINC00239 and Keap1, we used the RNAfold database to predict the secondary structure of LINC00239 (Fig. 3F). An in vitro RNA pull-down assay, followed by western blot analysis, confirmed that the 1–315 nt sequence of LINC00239 is essential for binding to Keap1 (Fig. 3G). Furthermore, protein domain deletion mutant studies demonstrated that LINC00239 interacts with the 322–609 amino acid (aa) region of Keap1 (Fig. 3H, I). Taken together, these findings suggest that Keap1 is a proven and novel binding partner of LINC00239.

### The LINC00239–Keap1 interaction in the cytoplasm is critical for the activation of the Nrf2 signaling pathway

**Fig. 4:**
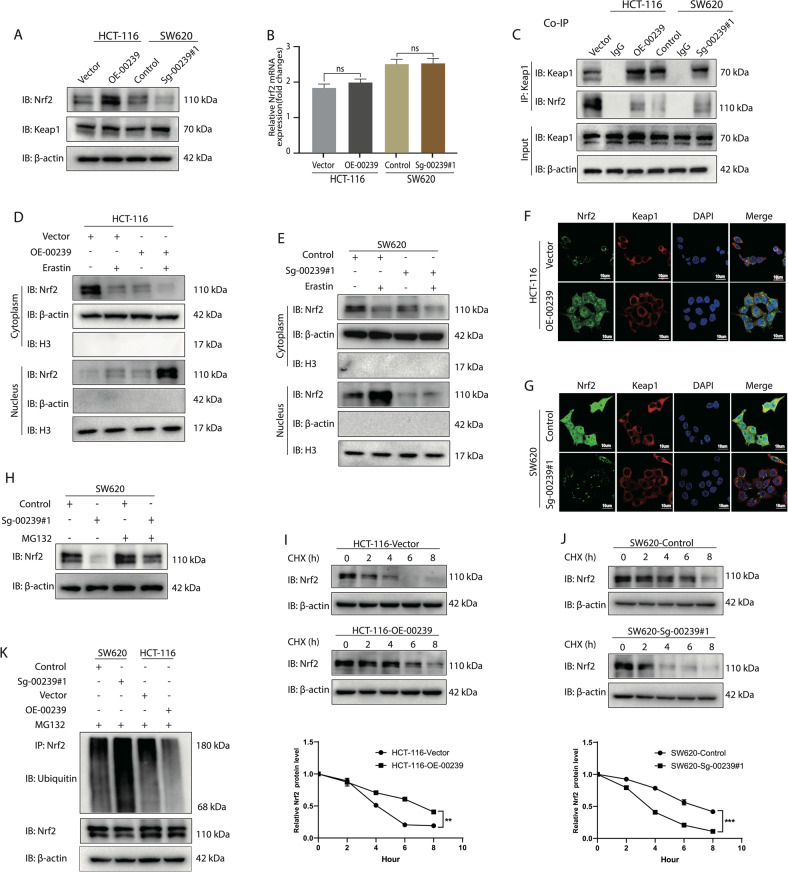
LINC00239 interacts with Keap1 and regulate the stability of Nrf2. **A** Western blot analysis of the expression levels of Keap1 and Nrf2. **B** The mRNA level of Nrf2 encoding gene (NFE2L2) was not significantly different in CRC cells lacking or overexpressing LINC00239. **C** LINC00239 reduced the interaction between Nrf2 and Keap1. CRC cells were treated with 10 μM erastin for 12 h. Cell lysates were immunoprecipitated with an anti-Keap1 antibody and blotted with an anti-Nrf2 antibody. **D**, **E** LINC00239 regulates the subcellular localization of Nrf2. Subcellular fractionation was used to isolate cytoplasmic and nuclear proteins, and immunoblotting was performed to examine the localization of Nrf2 following the downregulation or overexpression of LINC00239. **F**, **G** Immunofluorescence was used to localize Nrf2 in cells lacking or overexpressing LINC00239. All cells were treated with 10 μM erastin for 12 h. CRC cells were labeled with anti-Nrf2 (green), anti-Keap1 (red), and DAPI (blue). Scale bar: 10 μm. **H** LINC00239 reduced the protein degradation of Nrf2. SW620 cells transfected with LINC00239-knockdown and control plasmids were left untreated or treated with 10 μM of MG132 for 6 h to block the degradation of ubiquitinated proteins. **I**, **J** LINC00239 stabilized Nrf2 under basal conditions. All cells were left untreated or treated with 50 μg/mL CHX and incubated for the indicated time periods. Lysates were analyzed by western blotting. **K** LINC00239 reduced the ubiquitination of Nrf2. All cells treated with 10 μM of MG132 for 6 h and subjected to an in vivo ubiquitination assay to detect ubiquitin-conjugated endogenous Nrf2 proteins. Lysates were denatured, immunoprecipitated with anti-Nrf2 and blotted with an anti-ubiquitin antibody. Data shown represent mean ± SD from three independent experiments. ns *P* > 0.05, **P < 0.01, ***P < 0.001, ****P < 0.0001, Student’s *t* test.

Numerous regulators are known to have significant impacts on intracellular anti-ferroptosis defenses. NFE2-related factor 2 (Nrf2) is the key regulator of anti-ferroptosis. Keap1 brings Nrf2 into the Cul3-dependent E3 ubiquitin ligase complex through the Kelch domain, leading to the rapid proteasomal degradation of Nrf2 [36, 37]. Nrf2 can regulate a large number of downstream effectors against ferroptosis, including FTH1, GCLC, GCLM, HO-1, NQO1, and GPX4, and others, via binding enhancer sequences termed “antioxidant-response elements” (AREs) [38]. Interestingly, we found that LINC00239 interacts with the Kelch domain of the Keap1 protein (Fig. 3H, I). Therefore, we hypothesized that LINC00239 can inhibit the ubiquitination of Nrf2 by interacting with the Kelch domain. To determine whether these interactions could influence the stability of the Keap1/Nrf2 complex, we first examined the expression of Nrf2 and Keap1 in CRC by western blotting. The results revealed that although dysregulation of LINC00239 did not influence Keap1 expression, upregulation of LINC00239 significantly increased Nrf2 protein expression, whereas diminished expression of LINC00239 suppressed Nrf2 levels (Fig. 4A). In addition, dysregulation of LINC00239 did not affect the mRNA expression of Nrf2 (Fig. 4B), suggesting that the interaction between LINC00239 and Keap1 mainly leads to instability of the Keap1/Nrf2 complex. Co-IP experiments further validated that upregulation of LINC00239 inhibited the stability of the Keap1/Nrf2 complex, leading to decreased expression of Nrf2, and vice versa (Fig. 4C). Once Nrf2 dissociates from Keap1, it translocates into the nucleus. We next evaluated the expression and distribution of Nrf2. Western blot analysis revealed that overexpression of LINC00239 induced Nrf2 translocation from the cytoplasm to the nucleus, especially in the presence of erastin. Conversely, knockout of LINC00239 strongly inhibited Nrf2 translocation into the nucleus under erastin treatment (Fig. 4D, E). Immunofluorescence staining further confirmed that LINC00239 promotes Nrf2 expression and nuclear translocation (Fig. 4F, G). To determine whether dysregulation of LINC00239 affects ubiquitination and proteasomal degradation of Nrf2, we treated SW620-sg-00239#1 cells and the corresponding controls with the proteasome inhibitor MG132. Western blot analysis revealed that MG132 significantly restored Nrf2 protein levels in SW620-sg-00239#1 cells (Fig. 4H). Moreover, the Nrf2 protein half-life was obviously prolonged when LINC00239 was overexpressed, whereas the Nrf2 protein half-life was obviously decreased when LINC00239 was downregulated (Fig. 4I, J), suggesting that LINC00239 can regulate Nrf2 via its protein degradation. In addition, the polyubiquitination of Nrf2 was substantially decreased when LINC00239 was overexpressed, while Nrf2 ubiquitination was dramatically increased when LINC00239 was silenced (Fig. 4K).

Taken together, these data indicated that LINC00239 can stabilize Nrf2 by suppressing its ubiquitination in CRC. Previous studies have shown that the transcriptional activity of antioxidant-response elements (AREs) can reflect the activity of the Nrf2 signaling pathway [38]. To further verify that the anti-ferroptosis function of LINC00239 was mediated through the upregulation of Nrf2 signaling, we performed ARE luciferase reporter assays. The results showed that ARE luciferase expression was significantly reduced in LINC00239 knockdown cells (Supplementary Fig. 1D). Conversely, overexpression of LINC00239 significantly enhanced the expression level of ARE luciferase (Supplementary Fig. 1D). Simultaneously, we also examined the downstream effectors known to be regulated by Nrf2 and found that overexpression of LINC00239 induced the expression of FTH1, GCLC, GCLM, HO-1, NQO1, and GPX4, especially after exposure to erastin (Supplementary Fig. 1E). Conversely, knockout of LINC00239 inhibited FTH1, GCLC, GCLM, HO-1, NQO1, and GPX4 expression (Supplementary Fig. 1F). All these findings suggest that the dysregulation of LINC00239 affects the ubiquitination and proteasomal degradation of Nrf2.

### LINC00239 regulates ferroptosis by modulating the expression of Keap1/Nrf2 signaling pathway

**Fig. 5:**
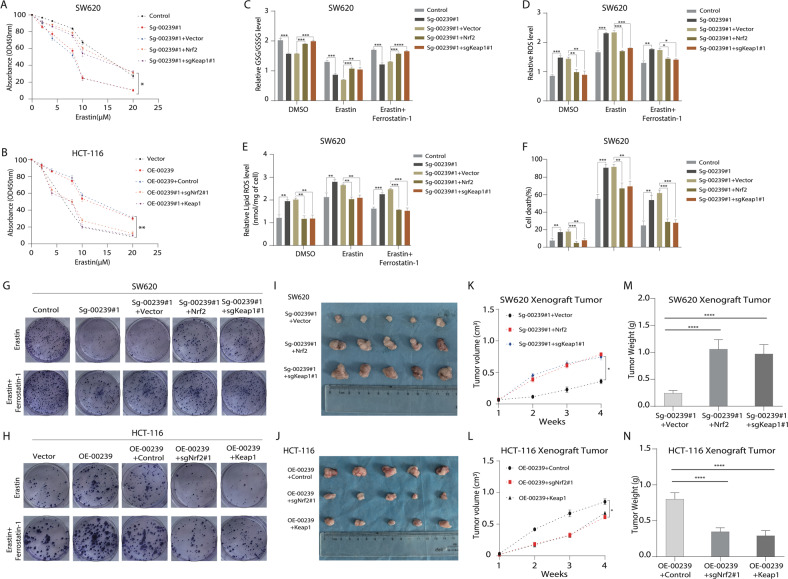
LINC00239 regulates ferroptosis by modulating the expression of the Keap1/Nrf2 signaling pathway. **A**, **B** Dose-dependent toxicity of erastin-induced cell death. **C**–**F** GSH/GSSG ratios (**C**), ROS levels (**D**), lipid ROS (**E**), and cell viability (**F**) was measured in the four indicated cell lines after treated with 10 μM erastin for 48 h and the addition of 2 µM ferrostatin-1 (Fer-1). **G**, **H** Colony-formation assay to evaluate the cell viability of LINC00239 on colorectal cancer cells after treated with 2 μM erastin and the addition of 2 µM ferrostatin-1 (Fer-1). **I**–**N** Nude mice are shown after injection of HCT-116-OE-00239 and SW620-sg-00239#1 cells stably expressing the control vector, sgNrf2#1, OE-Nrf2, sgKeap1#1, or OE-Keap1 expression plasmids. Tumor formation was monitored at the images (**I**, **J**), indicated times (**K**, **L**), and weights (**M**, **N**) were recorded (*n* = 5). All experiments were thereafter treated with erastin three times a week. Data shown represent mean ± SD from three independent experiments. ns *P* > 0.05, **P < 0.01, ***P < 0.001, ****P < 0.0001, Student’s *t* test.

To further demonstrate whether Keap1/Nrf2 signaling is involved in the ferroptosis-suppressing activity of LINC00239, we constructed HCT-116-OE-00239-Keap1, HCT-116-OE-00239-sgNrf2#1, SW620-sg-00239#1-sgKeap1#1, and SW620-sg-00239#1-Nrf2 cells and their control cells.(Supplementary Fig. 2). ML334 is a potent, cell-permeable NRF2 activator that acts by inhibiting the Keap1-NRF2 protein interaction [39]. Nrf2-IN-1 is an inhibitor of nuclear factor-erythroid 2-related factor 2 (Nrf2). Nrf2-IN-1 is developed for the research of acute myeloid leukemia (AML) [40]. Compared with the control treatment, knockout of Keap1 or treatment with the Keap1 inhibitor ML334, as well as overexpression of Nrf2 in SW620-sg-00239#1 cells, enhanced cell viability upon erastin exposure, whereas overexpression of Keap1, knockout of Nrf2 or treatment with the Nrf2 inhibitor Nrf2-IN-1 decreased cell viability under erastin conditions (Fig. 5A, B and Supplementary Fig. 4A, B), indicating that upregulation of Nrf2 or downregulation of Keap1 suppressed ferroptosis in the absence of LINC00239. Consistently, increased GSH/GSSG levels, reduced ROS accumulation, lipid peroxidation, accumulation of ferrous ions, percentage of cell death, and increased clonogenicity were also observed in SW620-sg-00239#1 cells with Keap1 knockout or treated with the Keap1 inhibitor ML334, as well as overexpression of Nrf2 under treatment with erastin, and this anti-ferroptotic effect could be further enhanced when the cells were simultaneously treated with erastin plus ferrostatin-1 (Fig. 5C–G and Supplementary Fig. 4C–F, K). Conversely, decreased GSH/GSSG levels, increased ROS accumulation, lipid peroxidation, accumulation of ferrous ions, and percentage of cell death and decreased clonogenicity were observed when Keap1 was overexpressed, when Nrf2 was knocked out or when HCT116-OE-LINC00239 cells were treated with the Nrf2 inhibitor Nrf2-IN-1 and erastin and this pro-ferroptotic effect was partly reversed when the cells were simultaneously treated with erastin plus ferrostatin-1 (Fig. 5H and Supplementary Figs. 3A–D and 4C–J, L). In addition, upregulation of Nrf2 or downregulation of Keap1 in SW620-sg-00239#1 cells induced FTH1, GCLC, GCLM, HO-1, NQO1, and GPX4 expression. Conversely, knockout of Nrf2 or upregulation of Keap1 suppressed FTH1, GCLC, GCLM, HO-1, NQO1, and GPX4 expression (Supplementary Fig. 3E, F).

In vivo tumorigenesis experiments also showed larger tumor sizes and heavier tumor weights in SW620-sg-00239#1-sgKeap1#1 and SW620-sg-00239#1-Nrf2 cells than in SW620-sg-00239#1 cells. In contrast, smaller tumor sizes and lighter tumor weights were observed in HCT116-OE-LINC00239-Keap1 and HCT116-OE-LINC00239-sgNrf2#1 cells, further confirming that Keap1/Nrf2 signaling is involved in the ferroptosis-suppressing activity of LINC00239 in CRC development (Fig. 5I–N). All these findings indicated that Keap1/Nrf2 signaling is the major mediator of the ferroptosis-suppressing activity of LINC00239.

### Nrf2 mediates positive feedback transcript activation of LINC00239 in CRC

**Fig. 6:**
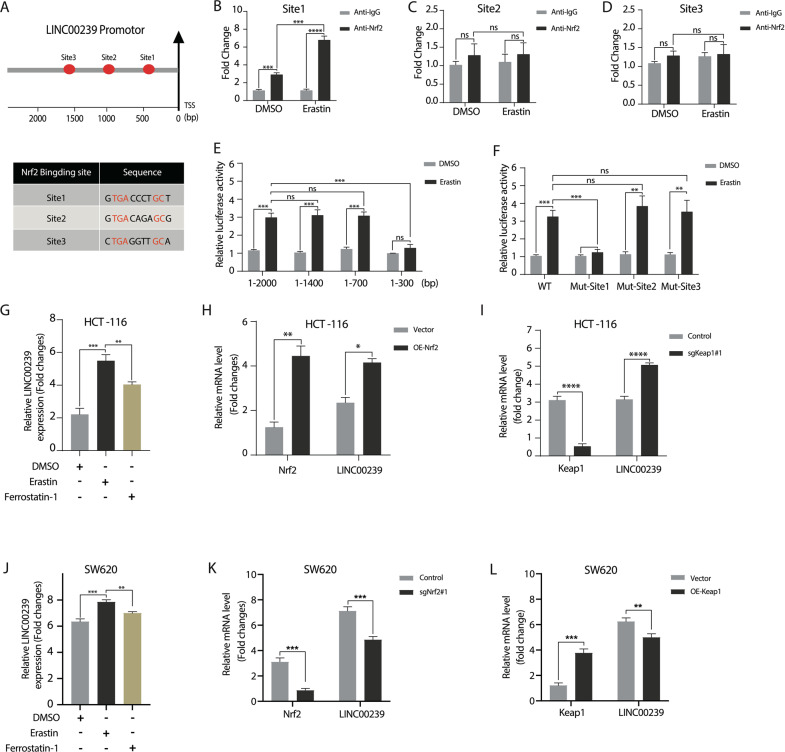
Nrf2 mediates positive feedback transcript activation of LINC00239 in CRC. **A** Schematic representation (upper panel) of the LINC00239 promoters from humans. Different symbols represent the Nrf2-binding sites. TSS transcriptional start site. **B**–**D** Chromatin immunoprecipitation (ChIP-qRT-PCR) analysis erastin increased the binding of Nrf2 to the LINC00239 promoter. SW620 cells were treated with DMSO or 10 μM erastin, and chromatin immunoprecipitation was performed using Nrf2-specific antibodies. **E**, **F** Erastin increased the binding of Nrf2 to the LINC00239 promoter. SW620 cells were transiently transfected with a luciferase reporter driven by the LINC00239 promoter (1–2000 bp, 1–1400 bp, 1–700 bp, or 1–300 bp), LINC00239 promoter (WT, mut-Site1, mut-Site2, or mut-Site3), and then were left untreated or treated with 10 μM erastin for 24 h. **G** HCT-116 cells were treated with DMSO, 10 µM erastin or 2 µM ferrostatin-1 (Fer-1) for 24 h. qPCR analysis the levels of LINC00239 in HCT-116 cells. **H** HCT-116 cells were transfected with control or Nrf2-overexpression plasmids. qPCR analysis the levels of LINC00239 and Nrf2 in HCT-116 cells. **I** HCT-116 cells were transfected with control or Keap1-knockdown (sgKeap1#1) plasmids. qPCR analysis the levels of LINC00239 and Keap1 in HCT-116 cells. **J** SW620 cells were treated with DMSO, 10 µM erastin, or 2 µM ferrostatin-1 (Fer-1) for 24 h. qPCR analysis the levels of LINC00239 in SW620 cells. **K** SW620 cells were transfected with control or Nrf2-knockdown (sgNrf2#1) plasmids. qPCR analysis the levels of LINC00239 and Nrf2 in SW620 cells. **L** SW620 cells were transfected with control or Nrf2-overexpression plasmids. qPCR analysis the levels of LINC00239 and Nrf2 in SW620 cells. Data shown represent mean ± SD from three independent experiments. ns *P* > 0.05, **P < 0.01, ***P < 0.001, ****P < 0.0001, Student’s *t* test.

Our above data show that LINC00239 can inhibit ferroptosis through the Keap1/Nrf2 signaling pathway. We previously found that erastin could enhance LINC00239 expression in CRC cells in vitro. However, we did not know how erastin influenced LINC00239 expression. To clarify the transcription factors that can mediate the expression of LINC00239 under erastin treatment, we analyzed the transcription factor binding sequence of the human LINC00239 gene promoter and found that LINC00239 contains three predicted Nrf2-targeting motifs in the promoter region (Fig. 6A). ChIP-qRT-PCR and luciferase reporter assays further demonstrated that the specific Nrf2-targeting sequence is located between 300 and 700 bp of the LINC00239 promoter region (Fig. 6B–E). In parallel to this result, mutation of the Nrf2 binding sites in this fragment decreased Nrf2-dependent activation of the LINC00239 reporter (Fig. 6F). To further investigate whether Nrf2 could positively regulate feedback transcript activation of LINC00239 in CRC, we treated CRC cells with erastin or ferrostatin-1, respectively, and detected the expression level of LINC00239 by real-time quantitative PCR. The results show that the expression level of LINC00239 can be increased under the treatment of erastin, and the expression level of LINC00239 can be decreased after adding ferrostatin-1 (Fig. 6G, J). In addition, we upregulated Nrf2 or downregulated Keap1 in HCT-116 cells. The results showed that direct upregulation of Nrf2 or knockout of Keap1, which promotes Nrf2 translocation into the nucleus, led to a significantly increased level of LINC00239 (Fig. 6H, I). Similarly, direct knockout of Nrf2 or overexpression of Keap1, which represses Nrf2 translocation into the nucleus, led to a significantly decreased level of LINC00239 (Fig. 6K, L). Overall, Nrf2 could mediate the positive feedback transcript activation of LINC00239 in CRC.

### LINC00239 expression has a positive correlation with Nrf2 and GPX4 expression in CRC tissues

**Fig. 7:**
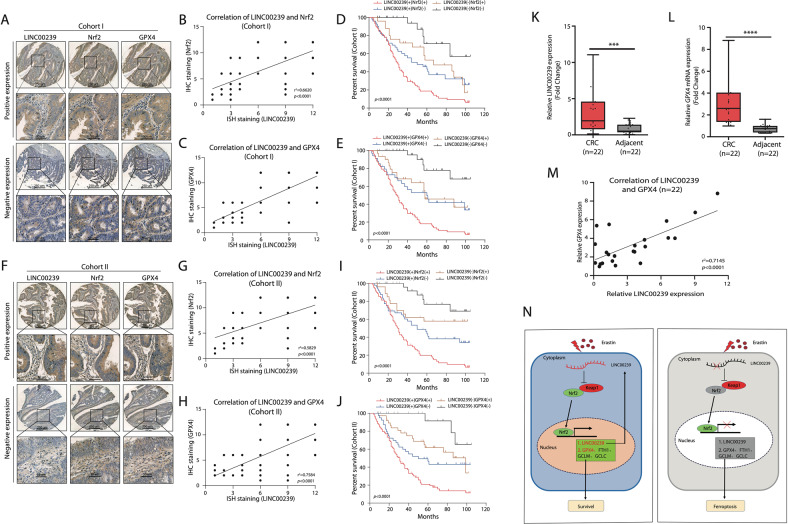
LINC00239 expression has a positive correlation with Nrf2 and GPX4 expression in CRC tissues. **A** Representative images of IHC or ISH staining of LINC00239, Nrf2, and GPX4 expression in human CRC samples in Cohort I (*n* = 174). The scale bars represent 250 μm (low magnification) and 50 μm (high magnification). **B** Correlation analysis of LINC00239 and Nrf2 expression in the CRC tissues in cohort I (*n* = 174). **C** Correlation analysis of LINC00239 and GPX4 expression in the CRC tissues in cohort I (*n* = 174). **D**, **E** Kaplan–Meier’s curves generated with the data from the CRC patients with negative versus positive LINC00239, Nrf2, or GPX4 expression. The correlation between LINC00239 and Nrf2 (D) or GPX4 (**E**) expression and overall survival or recurrence in patients with CRC in cohort I (*n* = 174). **F** Representative images of IHC or ISH staining of LINC00239, Nrf2, and GPX4 expression in human CRC samples in cohort II (*n* = 180). The scale bars represent 250 μm (low magnification) and 50 μm (high magnification). **G** Correlation analysis of LINC00239 and Nrf2 expression in the CRC tissues in cohort II (*n* = 180). **H** Correlation analysis of LINC00239 and GPX4 expression in the CRC tissues in cohort II (*n* = 180). **I**, **J** Kaplan–Meier’s curves generated with the data from the CRC patients with negative versus positive LINC00239, Nrf2 or GPX4 expression. The correlation between LINC00239 and Nrf2 (**I**) or GPX4 (**J**) expression and overall survival or recurrence in patients with CRC in cohort II (*n* = 180). **K**, **L** Real-time PCR of LINC00239 and GPX4 expression in adjacent nontumor samples and CRC samples (n = 22). **M** LINC00239 expression positively correlated with the expression levels of GPX4 in CRC samples (*n* = 22). **N** Proposed model of the relationship between LINC00239 and Nrf2 in CRC cells. Data shown represent mean ± SD from three independent experiments. ns *P* > 0.05, **P < 0.01, ***P < 0.001, ****P < 0.0001, Student’s *t* test.

The expression of LINC00239, Nrf2, and GPX4 were analyzed in two independent CRC cohorts. IHC and ISH staining that both Nrf2 and GPX4 showed a positive correlation with LINC00239 expression in two CRC cohorts (Fig. 7A–C, F–H). In addition, Kaplan–Meier analysis exhibited that CRC patients who had positive co-expression of either LINC00239/Nrf2 or LINC00239/GPX4 showed the lowest survival times in both CRC cohorts (Fig. 7D, E, I–J). To further investigate the roles of LINC00239 and Keap1/Nrf2 signal pathway in CRC, we detected the expression of LINC00239 and Nrf2 target gene (GPX4, GCLM, GCLC, FTH1, HO-1, and NQO1 in fresh CRC samples. The data revealed that LINC00239 is upregulated in CRC tissues compared with adjacent nontumor tissues and positively correlated with GPX4, GCLM, GCLC, FTH1, HO-1, and NQO1 levels (Fig. 7K–M and Supplementary Fig. 5). This work reveals a LINC00239-mediated ferroptosis regulatory mechanism in which oncogenic activation of the Keap1/Nrf2 signaling pathway suppresses ferroptosis in CRC. We confirmed that upregulation of LINC00239 promotes CRC proliferation by interacting with Keap1, causing instability of the Keap1/Nrf2 complex, thus enhancing Nrf2 protein stability and promoting CRC development. In turn, Nrf2 further promotes LINC00239 transcription, making LINC00239 a promising therapeutic strategy for CRC patients (Fig. 7N).

## Discussion

Ferroptosis, the novel form of non-apoptotic regulated cell death, is mainly caused by intracellular iron catalytic activity and lipid peroxidation, characterized by the accumulation of reactive oxygen species (ROS) [20, 41]. Recent studies have shown that excessive activation of iron death-related pathways can effectively prevent tumor progression and enhance the effects of targeted therapy, chemotherapy and even immunotherapy [16, 20, 42]. Regulation of pathways by which cells resist oxidative stress is critical for mitigating ferroptosis [35]. In particular, the glutathione pathway has been identified as a key antioxidant defense pathway. Central to this process is the metabolic protein glutathione peroxidase 4 (GPX4), which converts GSH to oxidized glutathione (GSSH), thereby protecting cells from iron by limiting cytotoxic lipid peroxidation deposition [43]. Antioxidant response elements (AREs) are found in the promoter regions of antioxidant genes in cells, and Nrf2 regulates gene expression by binding to the ARE sequences of cytoprotective genes [38]. The regulation of genes involved in oxidative stress, including GPX4, is largely controlled by the transcription factor Nrf2. It’s also a key defense against ferroptosis [44]. Normally, the expression of Nrf2 is inhibited by interacting with Keap1. Oxidative stress causes conformational changes in Keap1, disrupting this interaction, resulting in the stabilization of Nrf2 [38, 39]. Previous studies reported that p62/SQSTM1 can competitively inhibit the Keap1-Nrf2 complex, resulting in the upregulation of Nrf2 to resist ferroptosis [45].

LncRNAs are considered to be one of the key regulators of cancer progression and drug resistance by regulating the expression of downstream genes and various biological processes [19]. Accumulating evidence suggests that lncRNAs also play an important role in the occurrence of ferroptosis [46]. LncRNA p53RRA interacts with G3BP1 to promotes ferroptosis and apoptosis in lung cancer cells via nuclear sequestration of p53 [20]. Furthermore, lncRNA OIP5-AS1 inhibits ferroptosis by targeting the miR-128-3p/SLC7A11 pathway in prostate cancer [47]. A recent study found that the lncRNA NEAT1 promotes ferroptosis by regulating the miR-362-3p/MIOX axis in HCC cells [48]. LncRNAs play important roles in colorectal carcinogenesis, metastasis, and chemotherapy [49]. LINC00941 promotes CRC metastasis through preventing SMAD4 protein degradation and activating the TGF-β/SMAD2/3 signaling pathway [50]. In the present study, we found that lncRNAs also play an important role in ferroptosis in colorectal cancer.

In this study, we found that LINC00239 can promote the development of colorectal cancer by inhibiting ferroptosis. Previous studies have revealed that LINC00239, which is 652 nt in length, is upregulated in acute myeloid leukemia (AML), hepatocellular carcinoma (HCC), and CRC [51–53]. Yang et al.‘s study in acute myeloid leukemia found that LINC00239 could activate the PI3K/Akt/mTOR signaling pathway to increase the resistance of acute myeloid leukemia cells to chemotherapeutic drugs [52]. Their research clarified that LINC00239 can play a certain role in the regulation of tumor cell fate, but the specific mechanism of action is still unclear. Liang et al. found that LINC00239 could interact with C-Myc promoter-binding protein-1 (MBP-1) to promote the expression of the oncogene C-Myc in esophageal squamous cell carcinoma, thereby increasing the proliferation and metastatic capacity of ESCC cells [54]. Luo et al. showed that LINC00239 promotes colorectal cancer cell proliferation by sponging microRNA-484 and enhancing KLF12 expression [53]. These studies have well demonstrated that LINC00239 can regulate CRC progression by interacting with proteins. However, they still do not fully understand the mechanism of LINC00239 regulating ferroptosis in colorectal cancer cells.

In our study, we found that LINC00239 plays an important role in colorectal carcinogenesis. The recently discovered ferroptosis mode of programmed necrosis is a form of cell death that is independent of apoptosis. Ferroptosis is characterized by iron-dependent lethal accumulation of lipid ROS. Here, we demonstrate that LINC00239 decreases ROS, lipid ROS, and Fe^2+^ level, consistent with its role in ferroptosis. Moreover, the expression of several metabolic genes, including GPX4, which is linked to ferroptosis through its role in controlling lipid ROS, increased in the presence of LINC00239. Glutathione peroxidase 4 (GPX4) is a key regulator of ferroptosis. The expression of GPX4 is largely controlled by the transcription factor NRF2. LINC00239 inhibits ferroptosis in colorectal cancer by binding to Keap1 to stabilize Nrf2. Importantly, our work further proposed that Nrf2 also acted as an upstream regulator of LINC00239, thus forming a positive feedback loop to promote CRC development. To further address the clinical significance of LINC00239 in CRC, we evaluated LINC00239 expression in CRC tissue arrays and fresh CRC samples and found that a higher LINC00239 level was an independent risk factor for a poor prognosis in CRC patients (Fig. 7 and Supplementary Fig. 5).

In summary, this study suggested that LINC00239 promotes CRC proliferation by interacting with Keap1, causing instability of the Keap1/Nrf2 complex, thus enhancing Nrf2 protein stability and facilitating its nuclear translocation. Moreover, Nrf2 also acted as an upstream regulator of LINC00239, thus forming a positive feedback loop to promote CRC development, providing new insight into the mechanism of ferroptosis in CRC and a novel potential therapeutic target for advanced CRC.

## Supplementary information

Supplementary Figure Legends

Supplementary TableS1-S3

Supplementary Materials and methods

Springer Nature Reporting Summary

Original Date for Western blots

Supplementary Figure 1

Supplementary Figure 2

Supplementary Figure 3

Supplementary Figure 4

Supplementary Figure 5

Supplementary table S5

Supplementary Table S4

## Data Availability

The data that support the findings of this study are available from the corresponding author upon reasonable request.
