# Supplementary material for: Long noncoding RNA LINC00239 inhibits ferroptosis in colorectal cancer by binding to Keap1 to stabilize Nrf2
Source: Cell Death Dis. 2022 Aug 29;13(8):742. doi: 10.1038/s41419-022-05192-y (PMC9424287; doi:10.1038/s41419-022-05192-y)
Supplement: Supplementary file 1 — Supplementary Figure Legends [file 41419_2022_5192_MOESM1_ESM.docx]

**Supplementary Figure Legends**

**Supplementary Figure1. Characterization of LINC00239 in CRC. (A and B).** Determination of LINC00239 transcripts in SW620 cells by 5’ and 3’RACE. **(C).** qRT-PCR results of LINC00239 RNA level in SW620 cells after transfection with three independent LentiCRISPR-Cas13d-sgRNA(sg-00239#1, sg-00239#2, sg-00239#3) and Control. **(D).** A luciferase assay was used to detect reporter gene activity from the AREs. **(E and F).** The mRNA levels of Nrf2-downstream genes were analyzed by qPCR. HCT-116 cells transfected with LINC00239-overexpression and control plasmids (E). SW620 cells transfected with LINC00239-knockdown and control plasmids (F). All cells were left untreated or treated with 10 μM erastin for 24 h. Data shown represent mean ± SD from three independent experiments. ns *P*>0.05, ***P* < 0.01, ****P* < 0.001, *****P* < 0.0001, Student’s t test.

**Supplementary Figure2, relative to Figure 5. Nrf2/keap1 signal pathway participate in the biological function of LINC00239 in CRC cells. (A).** qRT-PCR results of Nrf2 mRNA level in SW620 cells after transfection with three independent LentiCRISPR-Cas13d-sgRNA(sgNrf2#1, sg-sgNrf2#2, sgNrf2#3) and Control. **(B).** Western blot results of Nrf2 protein level in SW620 cells after transfection with three independent LentiCRISPR-Cas13d-sgRNA(sgNrf2#1, sg-sgNrf2#2, sgNrf2#3) and Control. **(C).** qRT-PCR results of Keap1 mRNA level in SW620 cells after transfection with plvx-Keap1(OE-Keap1) and Vector. **(D).** Western blot results of Keap1 protein level in SW620 cells after transfection with plvx-Keap1(OE-Keap1) and Vector. **(E).** qRT-PCR results of Nrf2 mRNA level in HCT 116 cells after transfection with three independent LentiCRISPR-Cas13d-sgRNA(sgKeap1#1, sgKeap1#2, sgKeap1#3) and Control. **(F).** Western blot results of Keap1 protein level in HCT 116 cells after transfection with three independent LentiCRISPR-Cas13d-sgRNA (sgKeap1#1, sgKeap1#2, sgKeap1#3) and Control. **(G).** qRT-PCR results of Nrf2 mRNA level in HCT 116 cells after transfection with plvx-Nrf2(OE-Nrf2) and Vector. **(H).** Western blot results of Nrf2 protein level in HCT 116 cells after transfection with plvx-Nrf2 (OE-Nrf2) and Vector. **(I and J).** Western blot results of Nrf2 and Keap1 protein level in HCT 116 or SW620 cells. Data shown represent mean ± SD from three independent experiments. nsP>0.05, **P < 0.01, ***P < 0.001, ****P < 0.0001, Student’s t test.

**Supplementary Figure3, relative to Figure 5. Nrf2/keap1 signal pathway participate in the biological function of LINC00239 in CRC cells. (A-D).** GSH/GSSG ratios (A), ROS levels (B), Lipid ROS (C) and cell viability (D) was measured in the HCT-116 after treated with 10 μM erastin for 48 h and the addition of 2 µM ferrostatin-1 (Fer-1). **(E and F).** The mRNA levels of Nrf2-downstream genes were analyzed by qPCR. Data shown represent mean ± SD from three independent experiments. ns *P*>0.05, ***P* < 0.01, ****P* < 0.001, *****P* < 0.0001, Student’s t test.

**Supplementary Figure4, relative to Figure 5. Nrf2/keap1 signal pathway participate in the biological function of LINC00239 in CRC cells. (A-B).** Dose-dependent toxicity of Erastin-induced cell death of SW620-Control and SW620-sg-00239#1 cells were treated with DMSO or ML343, n=5 (A). Dose-dependent toxicity of Erastin-induced cell death of HCT 116-Vector and HCT 116-OE-00239 cells were treated with DMSO or Nrf2-IN-1, n=5 (B). **(C-F).** GSH/GSSG ratios (C), ROS levels (D), Lipid ROS (E) and cell viability (F) was measured in the HCT-116 after treated with 10 μM erastin for 48 h and the addition of 2 µM ferrostatin-1 (Fer-1). **(G-J).** GSH/GSSG ratios (G), ROS levels (H), Lipid ROS (I) and cell viability (J) was measured in the SW620 after treated with 10 μM erastin for 48 h and the addition of 2 µM ferrostatin-1 (Fer-1). **(K and L).** Colony-formation assay to evaluate the cell viability of LINC00239 on colorectal cancer cells after treated with 2 μM erastin and the addition of 2 µM ferrostatin-1 (Fer-1). Data shown represent mean ± SD from three independent experiments. ns *P*>0.05, ***P* < 0.01, ****P* < 0.001, *****P* < 0.0001, Student’s t test.

**Supplementary Figure 5, relative to Figure 7. LINC00239 expression has a positive correlation with Nrf2 and GPX4 expression in CRC tissues. (A-J).** LINC00239 expression positively correlated with the expression levels of Nrf2-target genes (GCLM, GCLC, FTH1, HO-1, and NQO1) in CRC specimens (n=22). qRT-PCR analysis of GCLM RNA levels in CRC specimens (n=22) **(A)**, LINC00239 expression positively correlated with the expression levels of GCLM in CRC specimens (n=22) **(B)**; GCLC mRNA levels in CRC specimens (n=22) **(C)**, LINC00239 expression positively correlated with the expression levels of GCLC in CRC specimens (n=22) **(D)**; FTH1 mRNA levels in CRC specimens (n=22) **(E)**, LINC00239 expression positively correlated with the expression levels of GCLC in CRC specimens (n=22) **(F)**; HO-1 mRNA levels in CRC specimens (n=22) **(G)**, LINC00239 expression positively correlated with the expression levels of HO-1 in CRC specimens (n=22) **(H)**; NQO1 mRNA levels in CRC specimens (n=22) **(I)**, LINC00239 expression positively correlated with the expression levels of NQO1 in CRC specimens (n=22) **(J)**. Data shown represent mean ± SD from three independent experiments. ns *P*>0.05, ***P* < 0.01, ****P* < 0.001, *****P* < 0.0001, Student’s t test.
