# Supplementary material for: Long noncoding RNA LINC00239 inhibits ferroptosis in colorectal cancer by binding to Keap1 to stabilize Nrf2
Source: Cell Death Dis. 2022 Aug 29;13(8):742. doi: 10.1038/s41419-022-05192-y (PMC9424287; doi:10.1038/s41419-022-05192-y)
Supplement: Supplementary file 2 — Supplementary TableS1-S3 [file 41419_2022_5192_MOESM2_ESM.docx]

| **siRNA sequences Sequence (5'-3')** | |
| --- | --- |
| Si-BBOX1-AS1#1 | TGAATACCAAAGAGGGCCGC |
| Si-BBOX1-AS1#2 | AATACCAAAGAGGGCCGCTG |
| Si-BBOX1-AS1#3 | CCATGGATGGGCACATTTGG |
| Si-LHFPL3-AS2#1 | CCTGGCAACCTGGCGGTTCC |
| Si-LHFPL3-AS2#2 | GGGTTGATGGAATCTAGTTT |
| Si-LHFPL3-AS2#3 | CACCTGGACTTTGGAAGCAG |
| Si-FOXD3-AS1#1 | AAGAGTAAGAGCAGCGCACC |
| Si-FOXD3-AS1#2 | ATTCCGGATGGACAGTGGTG |
| Si-FOXD3-AS1#3 | AAATCGGAAATGAGGCGCTG |
| Si-LINC00239#1 | CATTGGAAAGTGACGCACCC |
| Si-LINC00239#2 | ATTGGAAAGTGACGCACCCA |
| Si-LINC00239#3 | GGTACCAGCTGGGATGTTGG |
| Si-SH3PXD2A-AS1#1 | ACCGCGACTTGCTGAAAGAT |
| Si-SH3PXD2A-AS1#2 | CCTTGGCCCAAAACTTCACC |
| Si-SH3PXD2A-AS1#3 | CAGTGAAGCCCACGAACGTA |
| Si-LINC02532#1 | ACTGCTGGTTGGTAGCTGAG |
| Si-LINC02532#2 | AGGGAAAGCATCACCCGAAA |
| Si-LINC02532#3 | AGCCAGGACTCTTGGTAGGA |
| Si-FEZF1-AS1#1 | GATGCAGAGAGTGCTTGGGT |
| Si-FEZF1-AS1#2 | GTCACCTCGGTTCAGGTAGC |
| Si-FEZF1-AS1#3 | GCCTTAGGAGGCTTGTTCTGT |
| **SgRNA used in Cas13d** | **Sequence (5'-3')** |
| Sg-LINC00239#1 | TCGGTTGGCCCTTTCCTGCTTTA |
| Sg-LINC00239#2 | AAGACCTCCTTGAGTGCGGGCTG |
| Sg-LINC00239#3 | GAATACTCCCTGCAGCAAACAAG |
| Sg-Nrf2#1 | TGCCGATTCCTGCCCCTGCAGTC |
| Sg-Nrf2#2 | TTCAAGGCCATGTTCACCAACGG |
| Sg-Nrf2#3 | CTGGAAATGTGGTTCCCAGGGAT |
| Sg-Keap1#1 | TGCCGATTCCTGCCCCTGCAGTC |
| Sg-Keap1#2 | TTCAAGGCCATGTTCACCAACGG |
| Sg-Keap1#3 | CTGGAAATGTGGTTCCCAGGGAT |
| **Biotin labelled probes** | **Sequence (5'-3')** |
| sense-LINC00239 | ATTGGAAAGTGACGCACCCA |
| Antisense-LINC00239 | TGGGTGCGTCACTTTCCAAT |
| **FISH probe sequence of LINC00239 (5'-3')** | |
| RNA probe sequence | CCGCACACCTGCCTCATTGGAAAGTGACGCACCCACTGTTTGG  CCTCGGCAGCCTGATGTTTCTCATCACACAGGAAGACGGTCATG  CTACAGGAAGACCTCGGACTTTGCATC |

**Supplementary Table S1:** **All siRNA, sgRNA and probe information.**

**Supplementary Table S2:** **All primer information.**

| **Primer used in qRT-PCR** | | |
| --- | --- | --- |
| **Name** | **Sequence (5'-3')** | |
|  | **Forward** | **Reverse** |
| BBOX1-AS1 | CAGACTCCTGCTTTGCTCTT | GGAAGCATCTTCTCAGCTTCT |
| LHFPL3-AS2 | TCCTGGGGCGATGATATGGT | TGCTTCCAAAGTCCAGGTGC |
| FOXD3-AS1 | GAATAGTTGCCGAGAGAAA | GACAGACAGGGATTGGGTT |
| LINC00239 | AGGTACCAGCTGGGATGTTG | GGGTGCGTCACTTTCCAATG |
| SH3PXD2A-AS1 | GCCTCTTTACAGCGTTCCCT | CCTGAGGATACGTTCGTGGG |
| LINC02532 | CACTGCTGGTTGGTAGCTGA | CCCTCTCCCTCCCTTGACTT |
| FEZF1-AS1 | CGAACCGAGTCTGGACCATT | CTCCGCTCGGCATTCAGTAT |
| 18S | ACCCGTTGAACCCCATTCGTGA | GCCTCACTAAACCATCCAATCGG |
| Nrf2 | CACATCCAGTCAGAAACCAGTGG | GGAATGTCTGCGCCAAAAGCTG |
| Keap1 | CAACTTCGCTGAGCAGATTGGC | TGATGAGGGTCACCAGTTGGCA |
| FTH1 | TGAAGCTGCAGAACCAACGAGG | GCACACTCCATTGCATTCAGCC |
| GCLC | GGAAGTGGATGTGGACACCAGA | GCTTGTAGTCAGGATGGTTTGCG |
| GCLM | TCTTGCCTCCTGCTGTGTGATG | TTGGAAACTTGCTTCAGAAAGCAG |
| HO-1 | CCAGGCAGAGAATGCTGAGTTC | AAGACTGGGCTCTCCTTGTTGC |
| NQO1 | CCTGCCATTCTGAAAGGCTGGT | GTGGTGATGGAAAGCACTGCCT |
| GPX4 | ACAAGAACGGCTGCGTGGTGAA | GCCACACACTTGTGGAGCTAGA |
| **Primer used in ChIP-qRT-PCR** | | |
| **Name** | **Sequence (5'-3')** | |
|  | **Forward** | **Reverse** |
| LINC00239-Site1 | CCCCATTGTATTACCCCAGCAG | CACTCATCCATAACCACTG |
| LINC00239-Site3 | ATTGCGTGAACTTGGGAGGTC | ATTGCGTGAACTTGGGAGGTC |
| LINC00239-Site3 | AATTAGCCAGGTGTGTTGGCG | TTGACAGAGTCTCGCTCTGTAGCC |
| **Primers for *in vitro* transcription** | | |
| **Name** | **Sequence (5'-3')** | |
|  | **Forward** | **Reverse** |
| T7-LINC00239-sense (for in vitro transcription of F1) | taatacgactcactataTGCCTTTCACCTT  CTGCCATG | TCTCCCTTTGCAGACTGA |
| T7-LINC00239-antisense (for in vitro transcription of F0) | taatacgactcactataCATGGCAGAAGG  TGAAAGGCA | TCAGTCTGCAAAGGGAGA |
| T7-LINC00239-(1-315bp)-(for in vitro transcription of F1) | taatacgactcactataCATGGCAGAAGG  TGAAAGGCA | GTAGTGTGTCCTTCTGCCAGT |
| T7-LINC00239-(315-662bp)-(for in vitro transcription of F2) | taatacgactcactataCATCACACAGGAA  GACGGTCA | TCTCCCTTTGCAGACTGA |

**Supplementary Table S3:** **All antibody information.**

| **Name** | **Source** | **Identifiers** | **Additional information** |
| --- | --- | --- | --- |
| Anti-Keap1 | Proteintech | 10503-2-AP | WB (1:2000) |
| Anti-Keap1 | Proteintech | 60027-1-Ig | IF(1:50) |
| Anti-Keap1 | Proteintech | 10503-2-AP | IP (2ug for IP) |
| Anti-Nrf2 | Proteintech | 16396-1-AP | WB (1:1000) |
| Anti-Nrf2 | Proteintech | 16396-1-AP | IF(1:50) |
| Anti-Nrf2 | Proteintech | 16396-1-AP | IP (3ug for IP) |
| Anti-Nrf2 | Proteintech | 16396-1-AP | ChIP (5ug for ChIP) |
| Anti-Flag | Sigma-Aldrich | F1804 | WB (1:2000) |
| Anti-Flag | Sigma-Aldrich | F1804 | IP (5ug for IP) |
| Anti-Ubiquitin | Cell Signaling Technology | 3933S | WB (1:1000) |
| Anti-Histone H3 | Cell Signaling Technology | 14269S | WB (1:1000) |
| Anti-β-actin | Proteintech | 66009-1-Ig | WB (1:2000) |
| Goat Anti-Rabbit IgG-HRP | Abmart | M21002L | WB (1:2000) |
| Goat Anti-Mouse IgG HRP | Abmart | M21001L | WB (1:2000) |
| Dylight 594, Goat Anti-Mouse IgG | AntiProtech | SAG59401 | IF(1:100) |
| Dylight 488, Goat Anti-Rabbit IgG | AntiProtech | SAG48802 | IF(1:100) |
