# Supplementary material for: Long noncoding RNA LINC00239 inhibits ferroptosis in colorectal cancer by binding to Keap1 to stabilize Nrf2
Source: Cell Death Dis. 2022 Aug 29;13(8):742. doi: 10.1038/s41419-022-05192-y (PMC9424287; doi:10.1038/s41419-022-05192-y)
Supplement: Supplementary file 3 — Supplementary Materials and methods [file 41419_2022_5192_MOESM3_ESM.docx]

**Materials and methods**

**Measurement of cell death, cell viability, GSH/GSSG, ROS and lipid peroxidation**

Cell death was analysed by staining for SYTOX Green (Invitrogen, USA) followed by microscopy. The cell viability was evaluated using the alamarBlue cell viability test kit (Thermo Fisher Scientific, USA) according to the manufacturer's instructions. In short, cells were plated in 96-well plates and exposed to various concentrations of eaststin for the specified time. Add alamarBlue reagent to each well and incubate at 37°C, 5% CO_2_ for 6 hours, then measure the plate using a microplate reader (Thermo Fisher Scientific, USA) at 545 nm/590 nm (excitation/emission). The levels of GSH and GSSG are determined by the GSH and GSSG test kit (Beyotime, China) according to the manufacturer's procedures. The ROS level was determined using a commercial kit (Beyotime, China) according to the manufacturer's protocol. In short, cells were pre-incubated with DCFH-DA at 37°C for 30 minutes. After removing the extracellular dye, the cells were washed 3 times and incubated with serum-free DMEM. Subsequently, the fluorescence was measured using a microplate reader (Thermo Fisher Scientific, USA) at 488 nm excitation and 525 nm emission. Lipid Peroxidation Assay the relative malondialdehyde (MDA) concentration in cell lysates was assessed using a Lipid Peroxidation (MDA) Assay Kit (Abnova, China) according to the manufacturer’s instructions.

**RNA-seq**

The total RNA is separated into three biological replicates. We selected three pairs of colorectal cancer patient tissues. Patient #1: age (years): 59; sex: male; tumor location: right colon; tumor size: <5 cm; tumor invasion: T4; lymph node metastasis: absent; AJCC stage: Stage IV. Patient #2: age (years): 63; sex: male; tumor location: right colon; tumor size: <5 cm; tumor invasion: T3; lymph node metastasis: absent; AJCC stage: Stage III. Patient #3: age (years): 52; sex: male; tumor location: right colon; tumor size: <5 cm; tumor invasion: T4; lymph node metastasis: absent; AJCC stage: Stage IV.

Use TruSeq chain mRNA library preparation (Illumina, USA) for PolyA selection and cDNA library preparation. Paired-end 100 bp sequencing was performed on the Illumina HiSeq 4000 instrument. The RNA-seq read file was merged and analyzed from the technical copy, and Tophat was used to use the gencode annotation as a transcriptome index to locate it to the hg19 genome assembly. Use stringtie to assemble other transcripts and use HTSeq counting to count the reads in the exon sequence. Finally, EdgeR was used for differential expression analysis.

**Lentiviral packaging and infection**

The lentivirus packaging was performed following a previously established protocol^1^. Cell culture medium containing virus was collected 48 hours after transfection. Infected CRC cells were cultured in selective medium (medium containing 1.5 μg/ml puromycin) and collected 72 h after infection for downstream analysis. In order to carry out the rescue experiment, the overexpression virus was mixed with the knockdown virus before infecting the cells. For 5-aza treatment, 10 mM 5-aza stock solution in DMSO and freshly dilute with culture medium before use. Use a working concentration of 0.2μM 72 hours before collection, and refresh the 5-aza-containing medium every 24 hours. An equal amount of DMSO was added to the control cells.

**RNA pull-down assay**

Perform RNA affinity pull-down as previously described^2,3^. Briefly, the biotin-labeled RNA probe was transcribed in vitro according to the manufacturer's instructions (Pierce™ RNA 3' End Biotinylation Kit, Thermo Fisher Scientific, USA; in vitro Transcription T7 Kit, TaKaRa, Japan) and purified by Trizol (TaKaRa, Japan). Each pull-down uses 2 mg of purified biotinylated RNA. SW620 cells were resuspended in lysis buffer (10 mM Tris-HCl (pH 7.4), 100 mM NaCl, 2.5 mM MgCl_2_, 40 mg/ml digitalis saponins), and sonicated 3 times for 5s each time. Debris was removed by centrifugation at 12000 rpm for 15 minutes at 4°C. The cell lysate was then pre-purified by incubating with 40 ml streptavidin beads (Dyna-beads M 280 streptavidin, Invitrogen, USA) at 4°C for 2 hours. Inoculate the pre-clarified lysate with 2 mg of the biotinylated solution for 2 to 3 hours, and then incubate with the blocked beads overnight at 4°C. Then use high salt buffer (0.1% SDS, 1% Triton X-100, 2 mM EDTA, 20 mM Tris-HCl (pH 8.0), 500 mM NaCl), low salt buffer (0.1% SDS, 1% Triton) Wash beads X-100, 2 mM EDTA, 20 mM Tris-HCl (pH 8.0), 150 mM NaCl) and TE buffer. All buffers used in the previous steps contain RNase inhibitors, protease inhibitors and phosphatase inhibitors. The beads were then resuspended in SDS loading buffer and heated at 95°C for 5 minutes. The protein samples are then analyzed by mass spectrometry or western blotting.

**Mass spectrometry** **(LC-MS) analysis**

Perform mass spectrometry as previously described^3^. LC-MS analysis was performed on a Nano Aquity UPLC system (Waters Corporation, MA, US) connected to a quadrupole Orbitrap mass spectrometer (Q-Exactive) (Thermo Fisher Scientific, Bremen) equipped with an online nanoelectrospray ion source , Germany). Peptide samples were resuspended in 10 μl of solvent A (5% acetonitrile, 0.1% formic acid in water). Add 8 ul of sample to a Thermo Scientific Acclaim PepMap C18 column (100 μm × 2 cm, 5 μm, Thermo Fisher Scientific), set the flow rate to 10 μl/min for 3 min, and separate on the analytical column (Acclaim PepMap C18, 75 μm × 15 cm, 2 μm, 100 Å) with a linear gradient. The gradient started with 2% B (90% acetonitrile, 0.1% formic acid in water) over 75 minutes to 45% B. Re-equilibrate the column at initial conditions for 15 minutes. The column flow rate was maintained at 300 nL/min and the column temperature was maintained at 40 °C. Use an electrospray voltage of 2.2 kV relative to the mass spectrometer inlet. MS raw files generated by Q-Exactive were processed using MaxQuant software (version 1.5.2.8 http://www.maxquant.org/) for protein identification and quantification^4^. Data were searched against the Human UniProtKB/Swiss-Prot database using the Andromeda search engine^5^. The parameters are set as follows: (1) The minimum peptide length required is seven amino acids. (2) Apply trypsin cleavage specificity, allowing up to two missed cleavages. (3) The initial mass deviation of precursor and fragment ions is as high as 10 ppm and 0.5 Da, respectively. (4) The false discovery rate (FDR) at both the peptide and protein levels was set to 1%. (5) The minimum peptide segment is set to 5.

**RNA immunoprecipitation**

The RNA immunoprecipitation was performed following a previously established protocol^6^. HCT-116 wild-type cells from three 10 cm plates were fixed with 1% formaldehyde in PBS, fixed at room temperature for 10 minutes, then added with a final concentration of 100 mM glycine, and incubated at room temperature for 5 minutes. After washing 3 times with pre-cooling PBS, the cells were resuspended in 500ul RIP lysis buffer (1% SDS, 10 mM EDTA, 50 mM Tris-HCl pH8.1, supplemented with protease inhibitor cocktail and RNAase inhibitor), and Rotate for 30 minutes at 4°C. The lysate is then sonicated. The sonicated lysate was centrifuged at 4°C, 12000 rpm for 10 minutes to remove debris. Then transfer the supernatant, use RIP buffer (0.01% SDS, 1.1% Triton X-100, 1.2 mM EDTA, 16.7 mM Tris-HCl, pH 8.1, 167 mM NaCl) to make up to 1 ml, add 50 ml of pre-washed The Protein A/G Agarose beads (Thermo Fisher Scientific, USA) were incubated at 4°C with rotation for 1 hour to pre-clear the lysate. After pre-clearing, centrifuge at 10000 rpm for 5 minutes and transfer the supernatant. Take 100ul as Input, 450 ul for IgG, and 450 ul for IP (Anti-Keap1). Incubate overnight at 4°C with rotation. On the next day, add pre-washed agarose beads and incubate with rotation at 4°C for 2 hours. Then wash the beads 3 times with RIP buffer. Add 500ul Trizol (TaKaRa, Japan) to each sample. Follow the manufacturer's instructions for RNA isolation.

**Luciferase reporter assay**

The expression plasmid pGL3-WT and other related pGL3-linc00239 promoter vectors were transfected with 1.0 μg and 100 ng phRL (renilla luciferase). In our study, the mutant plasmids of the luciferase reporter system in Figure 6F were all chemically synthesized. pGL3-linc00239-mut-site1 mutates site1(GTGACCCTGCT) to mut-site1(GAAATTTTATT). pGL3-linc00239-mut-site2 mutates site2(GTGACAGAGCG) to mut-site2(GATATTTAGGG). pGL3-linc00239-mut-site3 mutates site3(CTGAGGTTGCA) to mut-site3(CTTATTAACCC).

Then, twenty-four hours after transfection, the cells were collected using the dual luciferase reporter gene detection system (Promega, USA) to detect luciferase activity. Luciferase activity was measured by using microplate reader (Thermo Fisher Scientific, USA). The transfection efficiency was normalized by dividing the luciferase activity of the construct by the corresponding Renilla luciferase activity^7^.

**Cell proliferation**

The cell proliferation was performed following a previously established protocol^8^. Cell proliferation was evaluated by Cell Counting Kit-8 (Dojindo, Japan). In short, control and treated colorectal cancer cells were seeded on 96-well plates at an initial density of 2×10^3^ cells per well. Add CCK-8 (10 μl/well) to the cells at the specified time point. After 2 hours of incubation, the reaction products were quantified according to the manufacturer's instructions^9^.

**Colony formation**

The colony formation was performed following a previously established protocol^10^. The cells were seeded in a 6-well plate (1×10^3^ cells/well), fixed and stained with crystal violet after 14 days.

**Generation of 3D spheroids**

The 3D spheroids were performed following a previously established protocol^11^. 3D collagen cultures were set up using 3 layers of type-I collagen PureCol (Advanced BioMatrix, USA) in triplicate as previously described.

1 Yi, W. *et al.* CRISPR-assisted detection of RNA-protein interactions in living cells. *Nature methods* **17**, 685-688, doi:10.1038/s41592-020-0866-0 (2020).

2 Wu, N. *et al.* LINC00941 promotes CRC metastasis through preventing SMAD4 protein degradation and activating the TGF-β/SMAD2/3 signaling pathway. *Cell death and differentiation* **28**, 219-232, doi:10.1038/s41418-020-0596-y (2021).

3 Tang, J. *et al.* LncRNA GLCC1 promotes colorectal carcinogenesis and glucose metabolism by stabilizing c-Myc. *Nature communications* **10**, 3499, doi:10.1038/s41467-019-11447-8 (2019).

4 Cox, J. & Mann, M. MaxQuant enables high peptide identification rates, individualized p.p.b.-range mass accuracies and proteome-wide protein quantification. *Nature biotechnology* **26**, 1367-1372, doi:10.1038/nbt.1511 (2008).

5 Cox, J. *et al.* Andromeda: a peptide search engine integrated into the MaxQuant environment. *Journal of proteome research* **10**, 1794-1805, doi:10.1021/pr101065j (2011).

6 Hao, Q. *et al.* SUNO1The S-phase-induced lncRNA promotes cell proliferation by controlling YAP1/Hippo signaling pathway. *eLife* **9**, doi:10.7554/eLife.55102 (2020).

7 Jiang, M. *et al.* O-GlcNAcylation promotes colorectal cancer metastasis via the miR-101-O-GlcNAc/EZH2 regulatory feedback circuit. *Oncogene* **38**, 301-316, doi:10.1038/s41388-018-0435-5 (2019).

8 Han, Y. *et al.* Long non-coding RNA MYOSLID functions as a competing endogenous RNA to regulate MCL-1 expression by sponging miR-29c-3p in gastric cancer. *Cell proliferation* **52**, e12678, doi:10.1111/cpr.12678 (2019).

9 Wu, N. *et al.* O-GlcNAcylation promotes colorectal cancer progression by regulating protein stability and potential catcinogenic function of DDX5. *Journal of cellular and molecular medicine* **23**, 1354-1362, doi:10.1111/jcmm.14038 (2019).

10 Wu, N. *et al.* miR-5590-3p inhibited tumor growth in gastric cancer by targeting DDX5/AKT/m-TOR pathway. *Biochemical and biophysical research communications* **503**, 1491-1497, doi:10.1016/j.bbrc.2018.07.068 (2018).

11 Lu, Y. *et al.* lncRNA MIR100HG-derived miR-100 and miR-125b mediate cetuximab resistance via Wnt/β-catenin signaling. *Nature medicine* **23**, 1331-1341, doi:10.1038/nm.4424 (2017).
