# Supplementary material for: Long noncoding RNA LINC00239 inhibits ferroptosis in colorectal cancer by binding to Keap1 to stabilize Nrf2
Source: Cell Death Dis. 2022 Aug 29;13(8):742. doi: 10.1038/s41419-022-05192-y (PMC9424287; doi:10.1038/s41419-022-05192-y)

Western Blot: Extended Data Fig. 3C

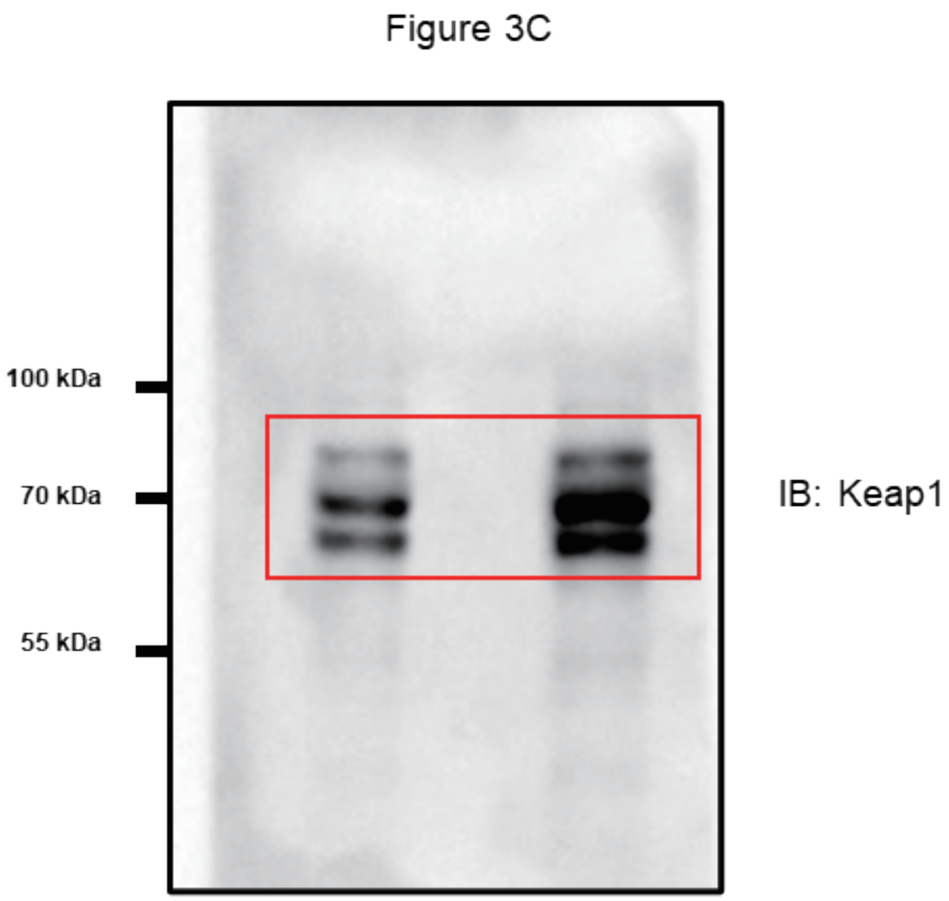

Gel Electrophoresis: Extended Data Fig. 3D

Figure 3D

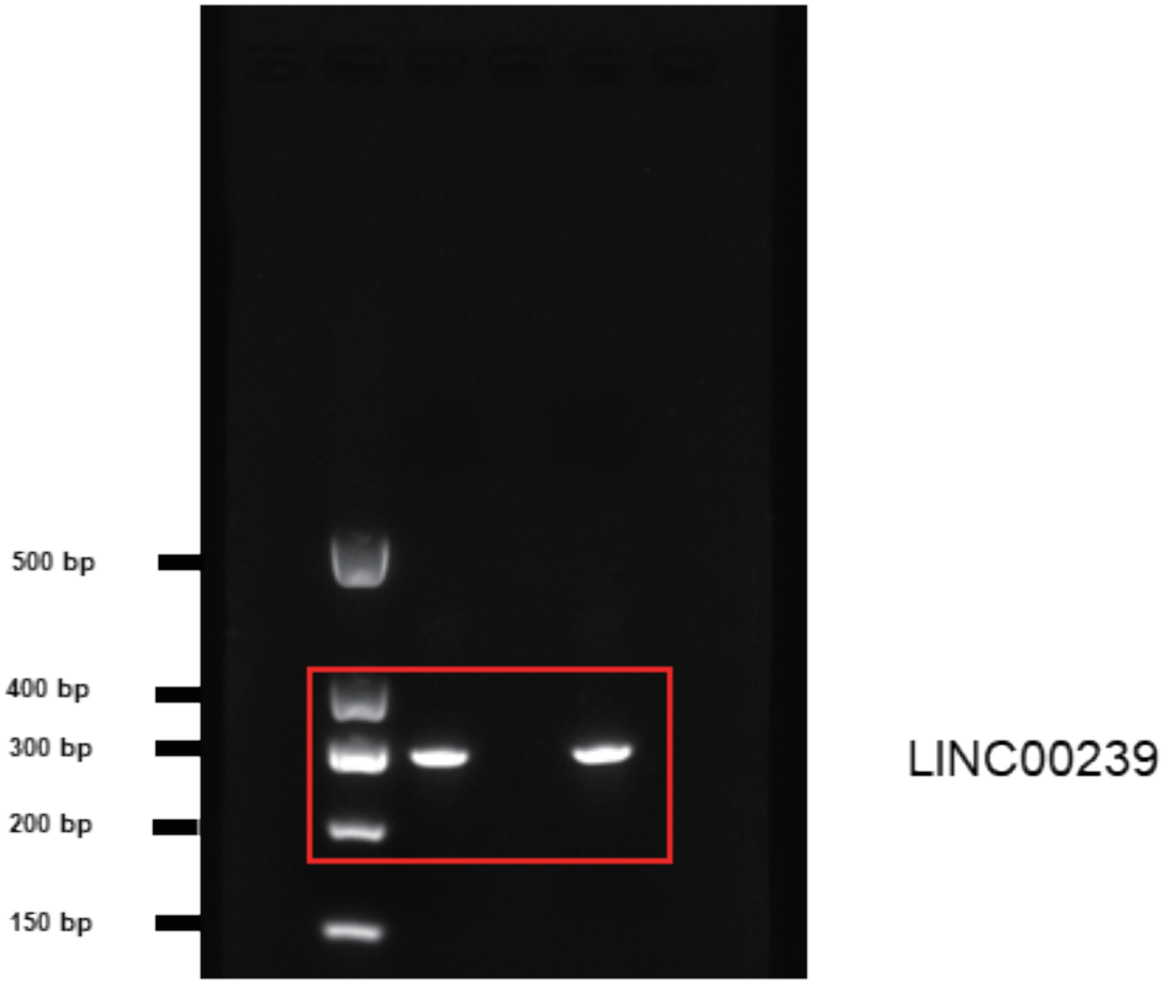

Gel Electrophoresis: Extended Data Fig. 3F

Figure 3F

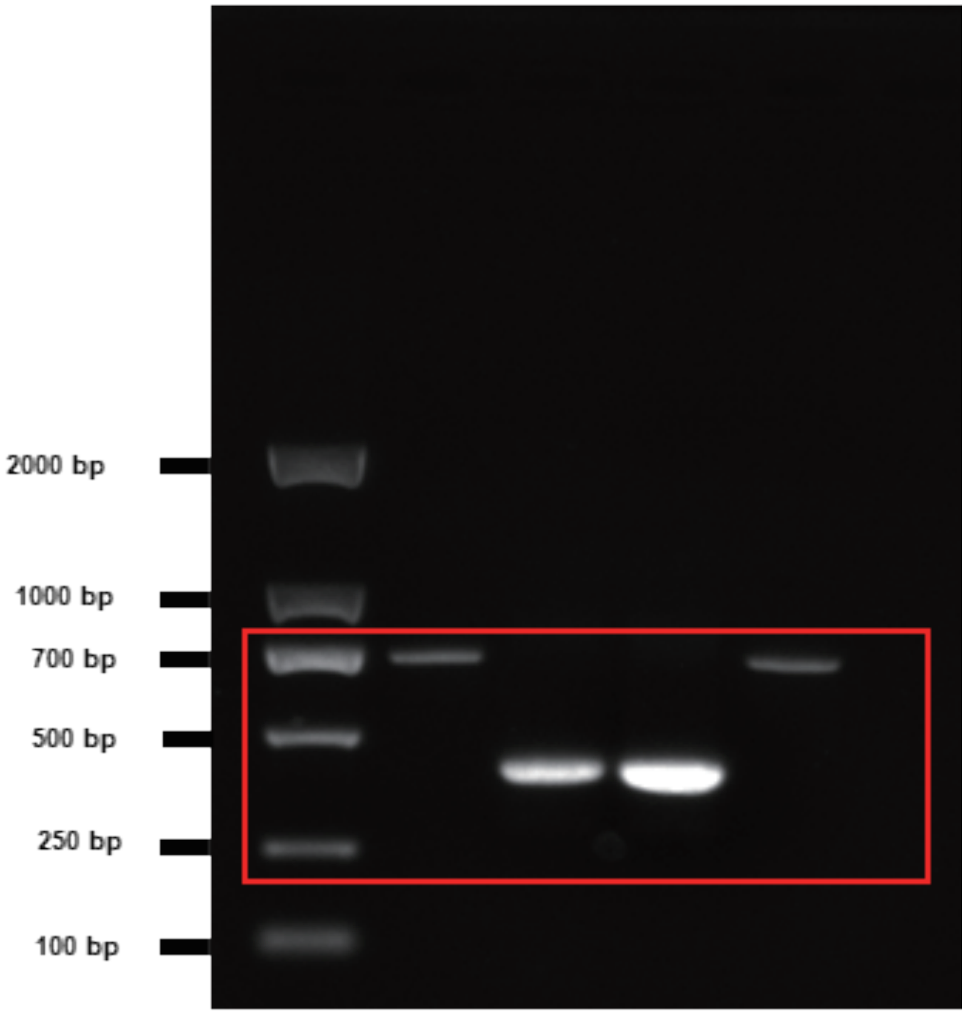

Western Blot: Extended Data Fig. 3G

Figure 3G

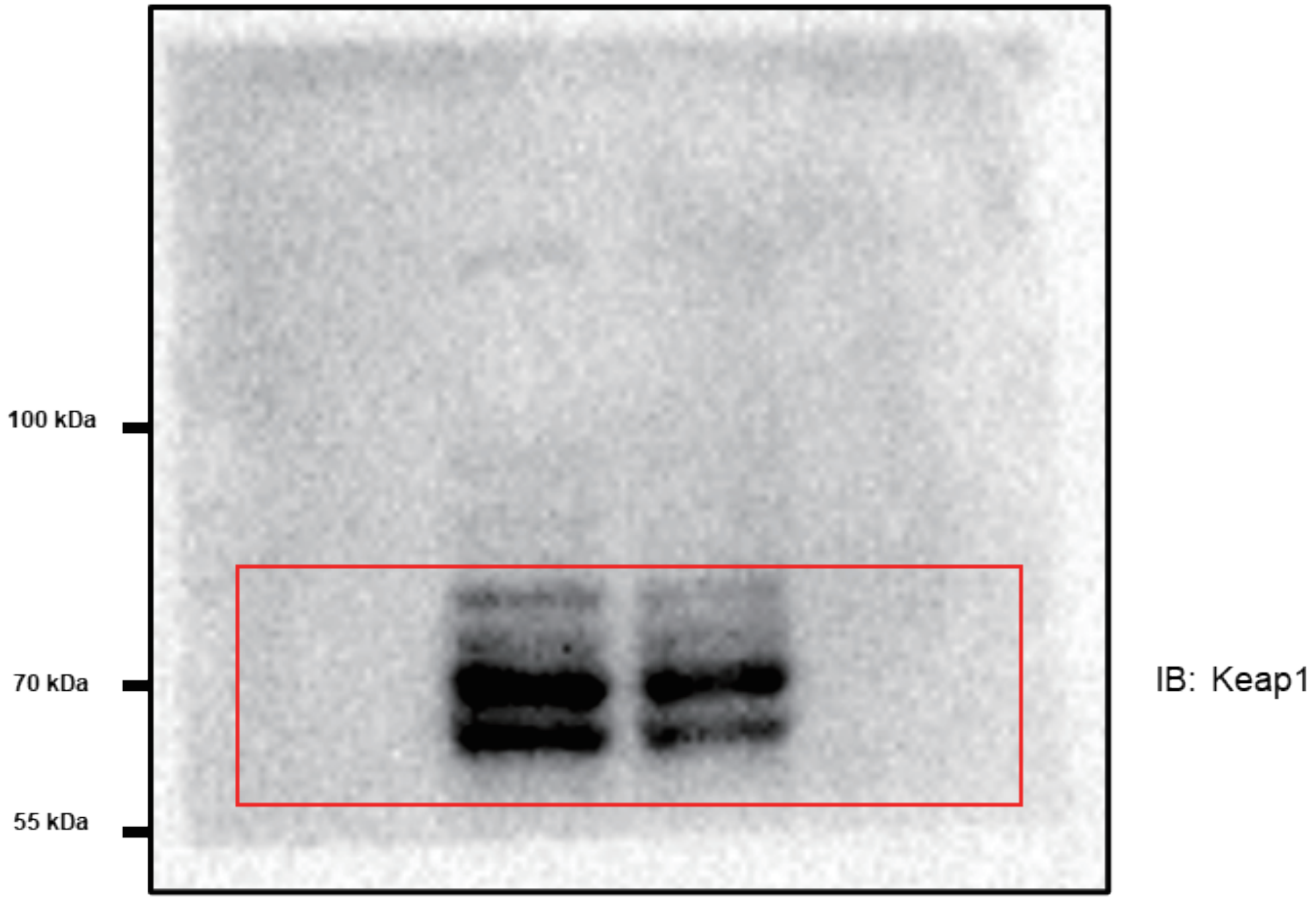

Western Blot: Extended Data Fig. 3H

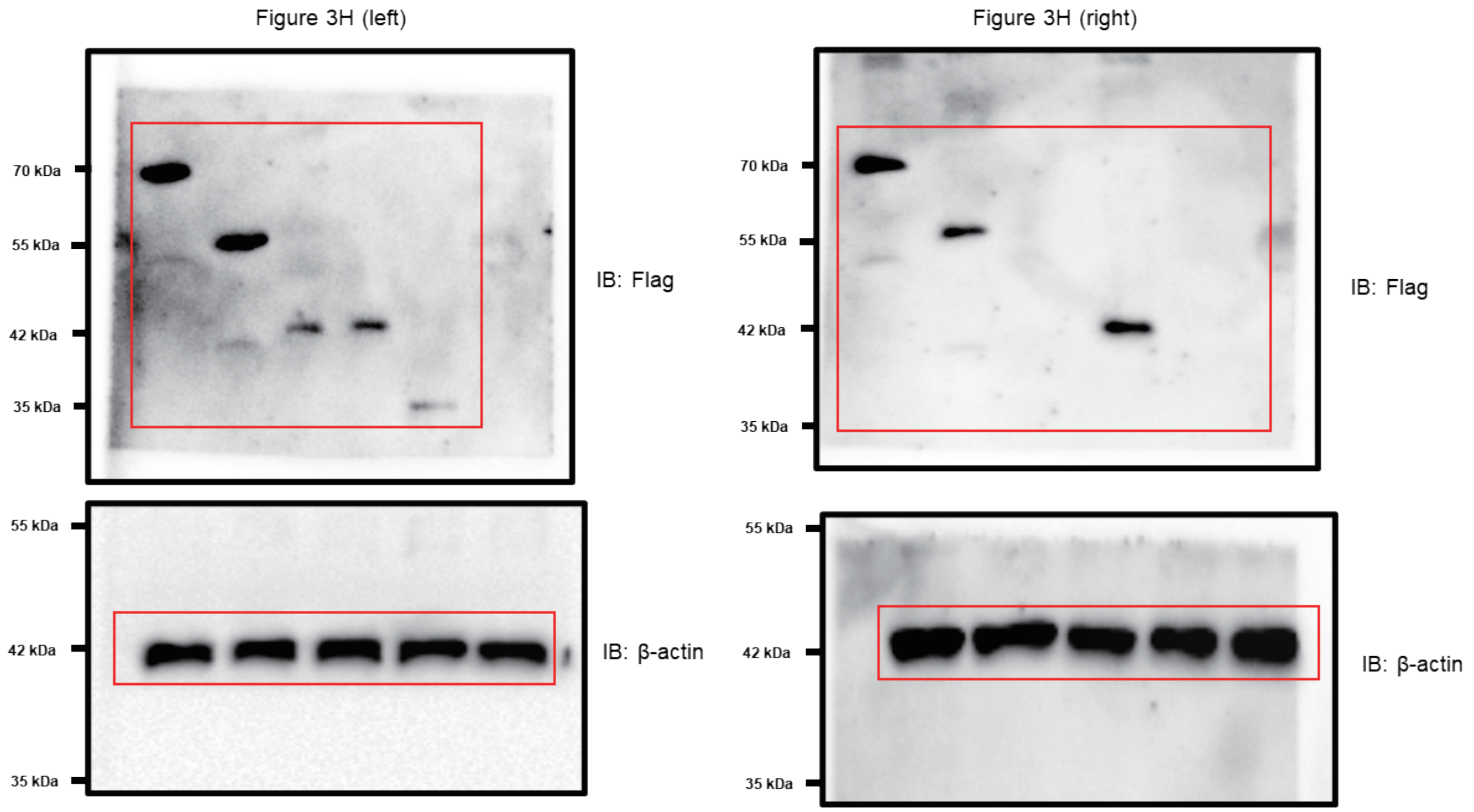

Western Blot: Extended Data Fig. 4A

Figure 4A

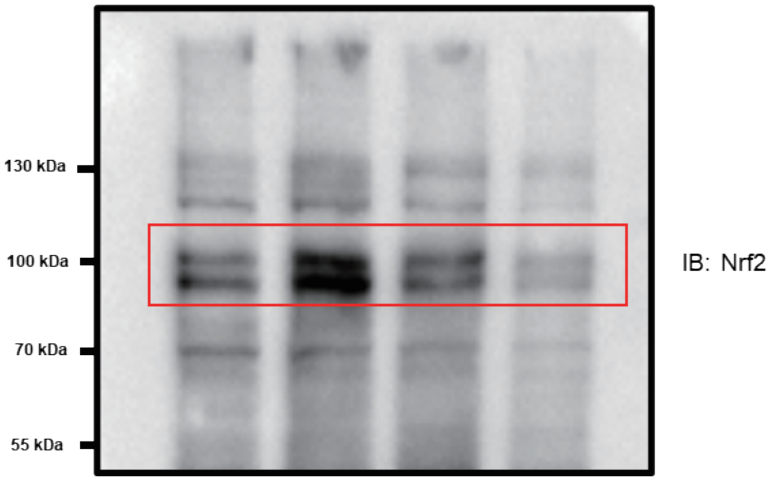

Figure 4A

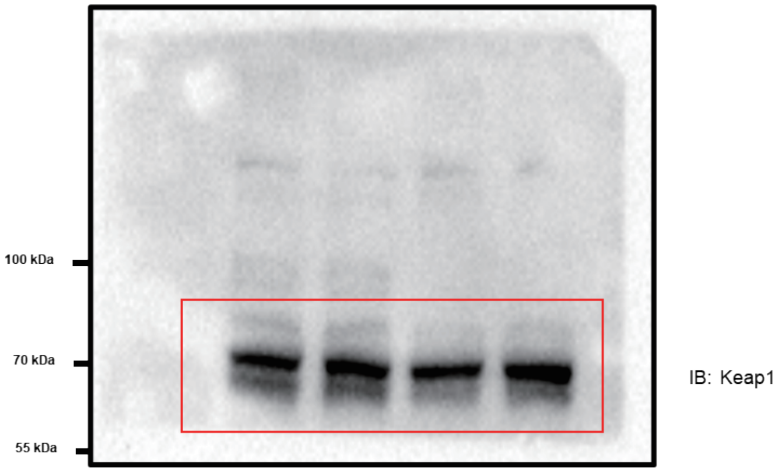

Figure 4A

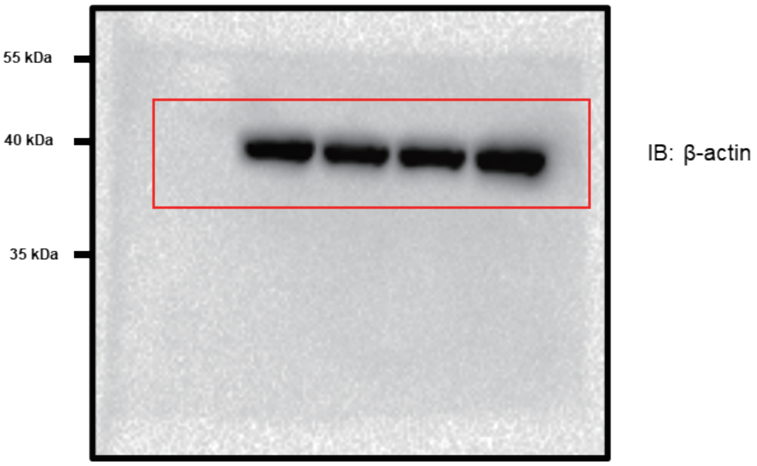

Western Blot: Extended Data Fig. 4C

Figure 4C

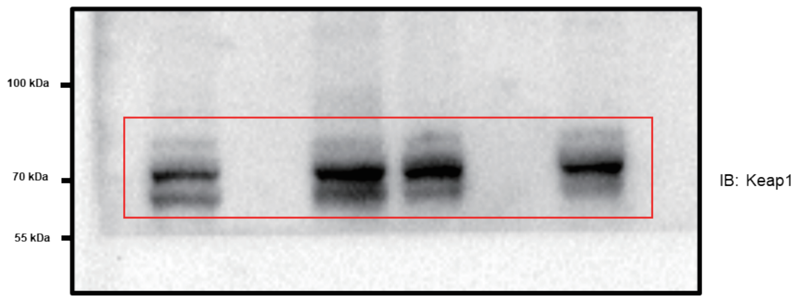

Figure 4C

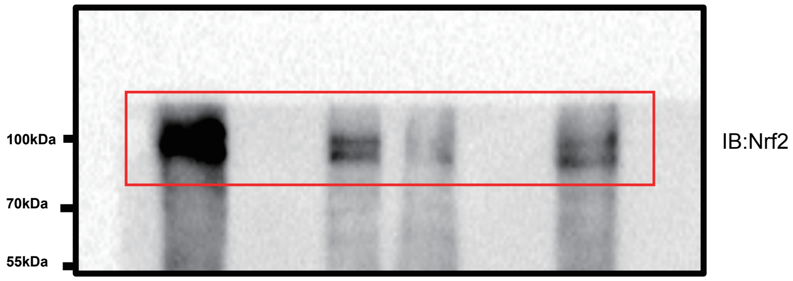

Figure 4C

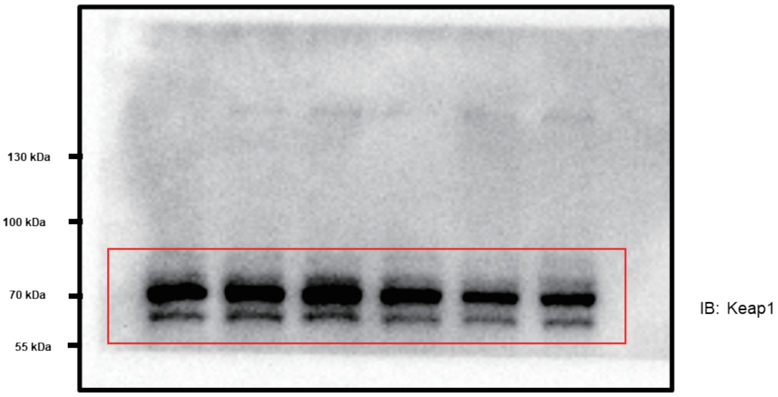

Figure 4C

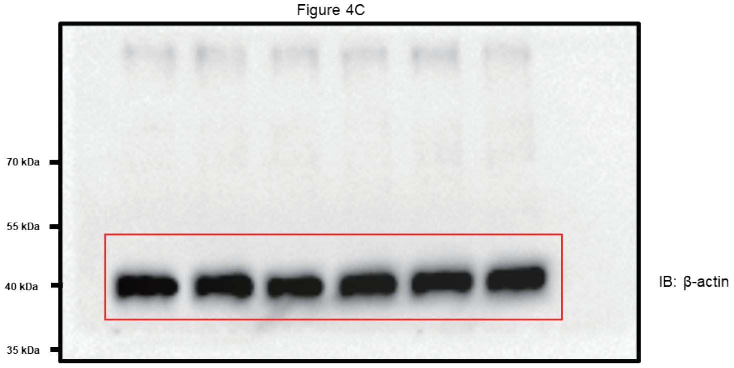

Western Blot: Extended Data Fig. 4D

Figure 4D

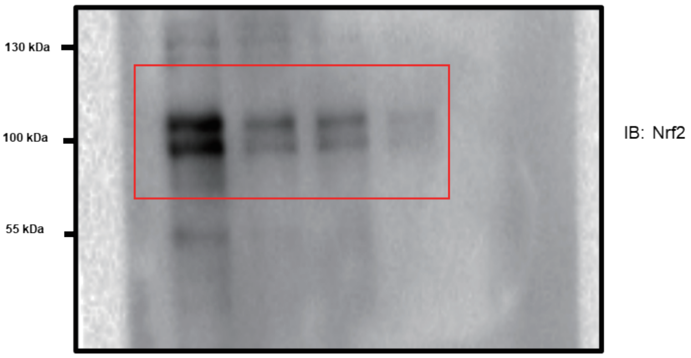

Figure 4D

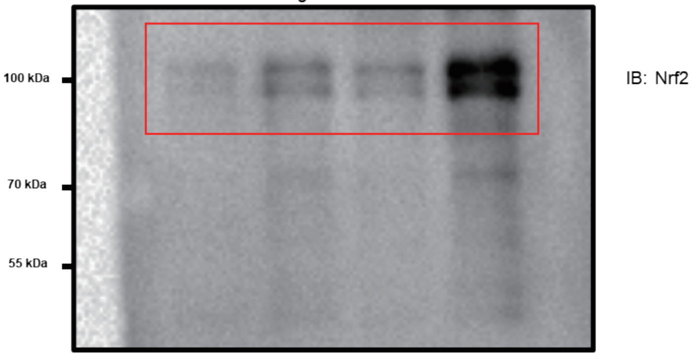

Figure 4D

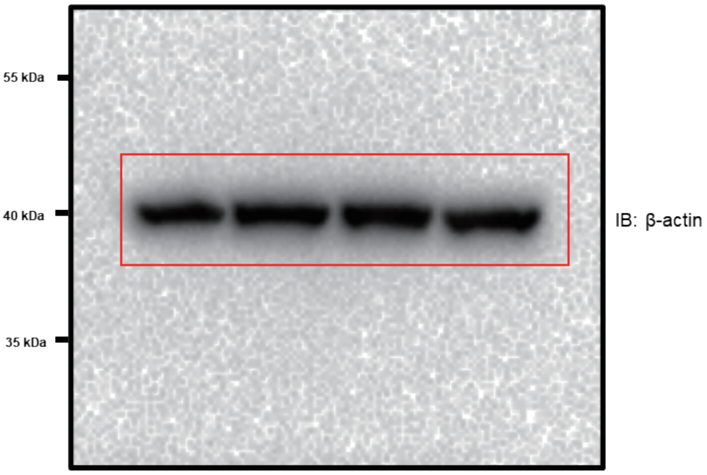

Figure 4D

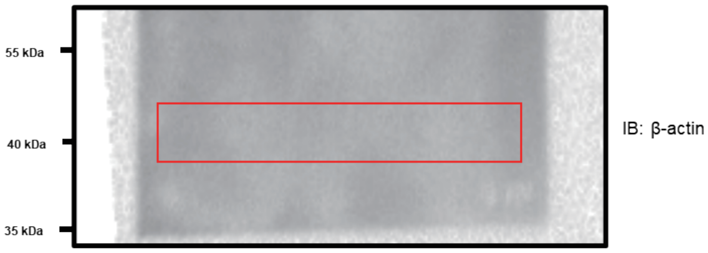

Figure 4D

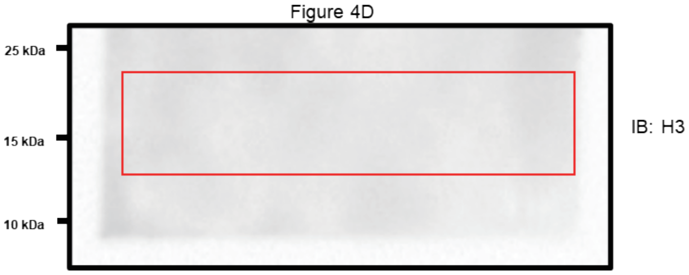

Figure 4D

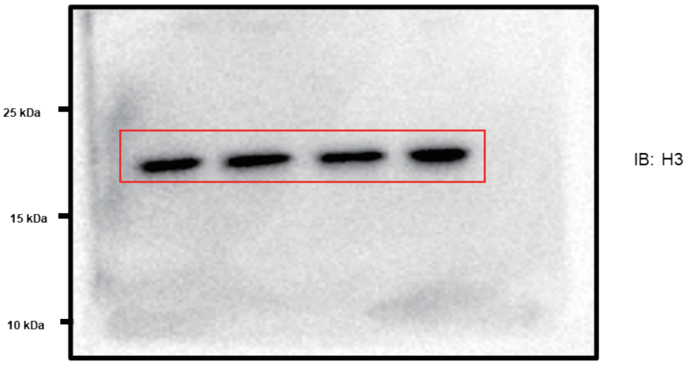

Western Blot: Extended Data Fig. 4E

Figure 4E

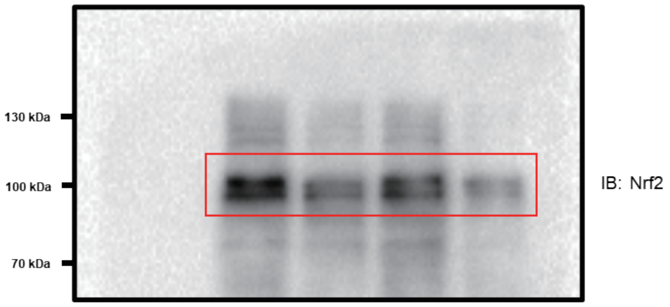

Figure 4E

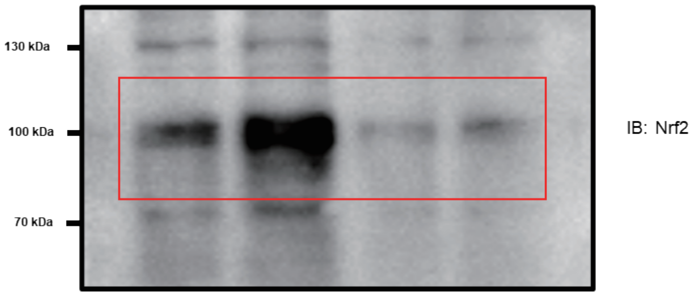

Figure 4E

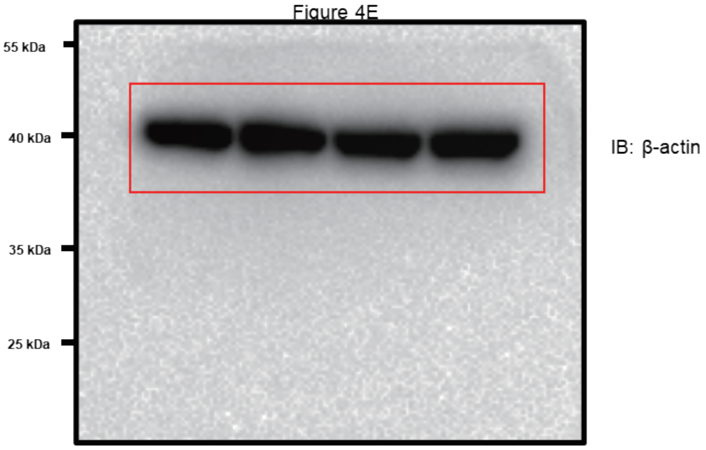

Figure 4E

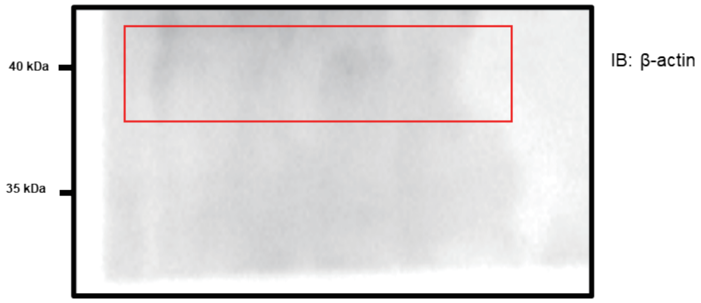

Figure 4E

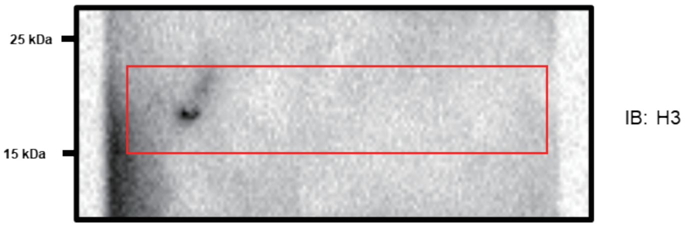

Figure 4E

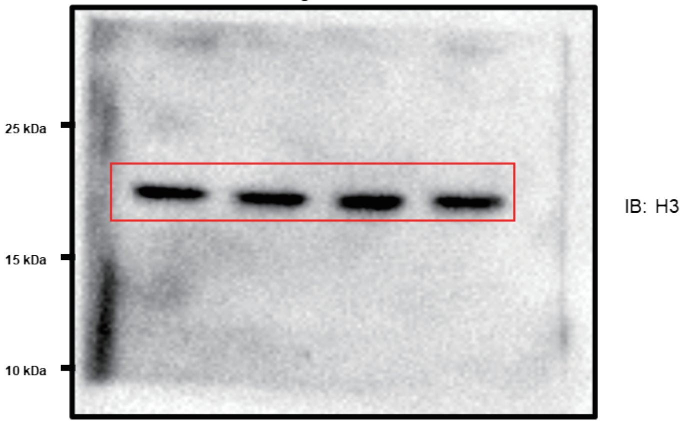

Western Blot: Extended Data Fig. 4H

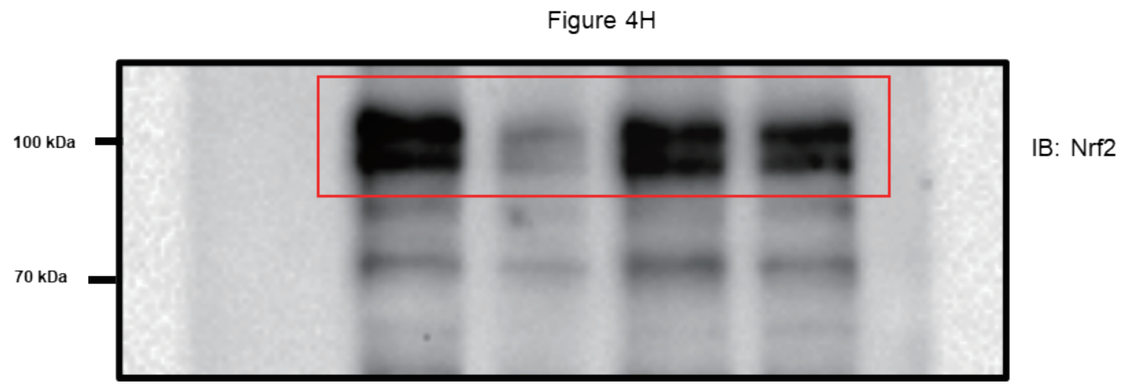

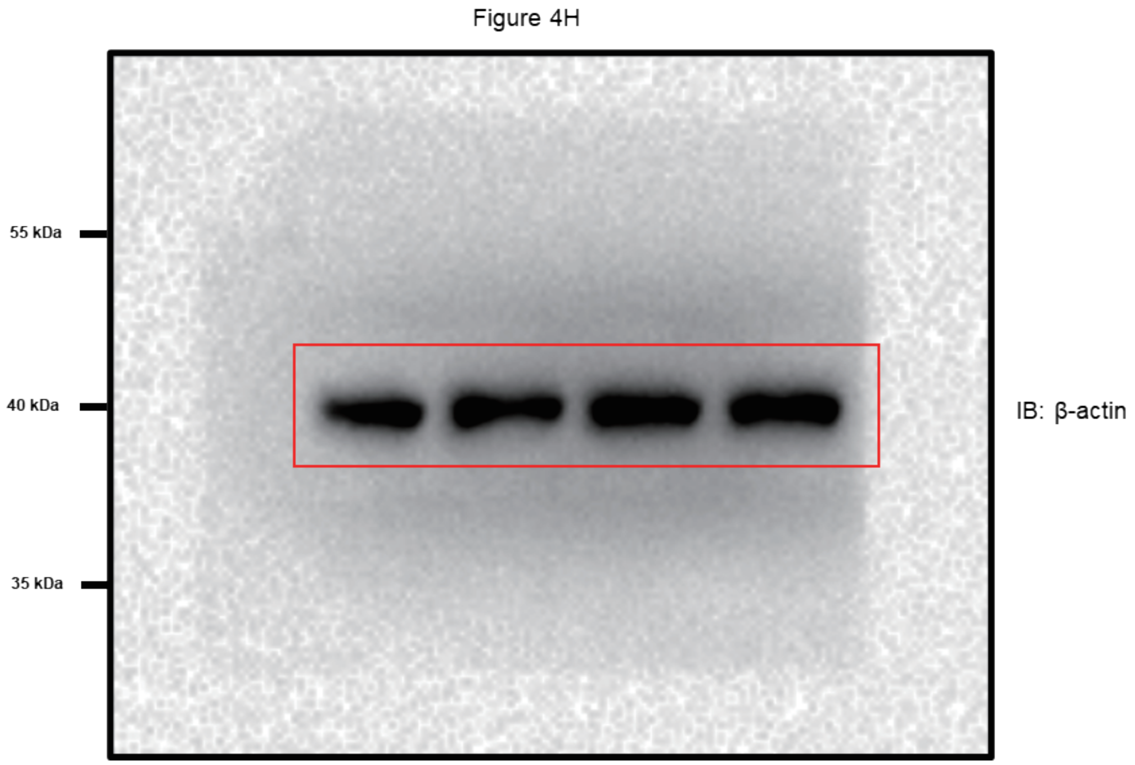

Western Blot: Extended Data Fig. 4l

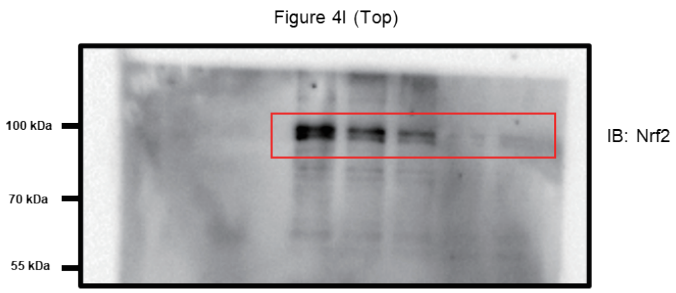

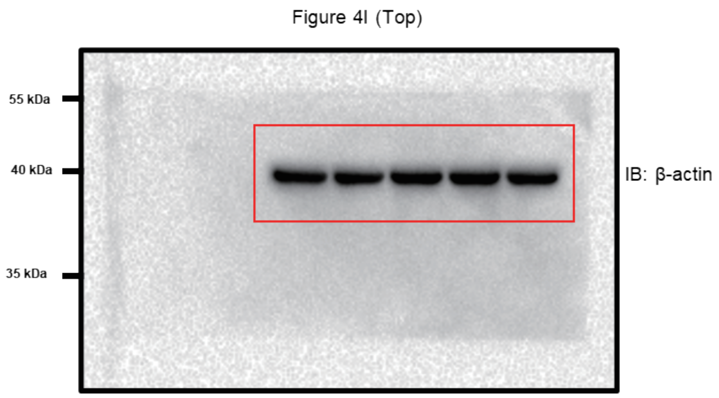

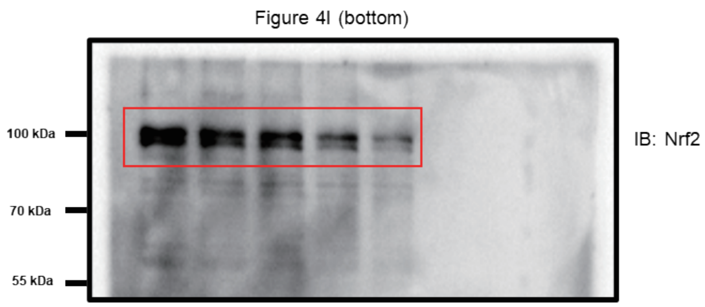

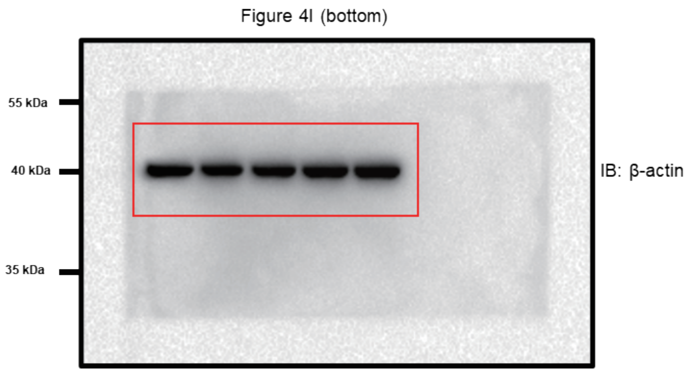

Western Blot: Extended Data Fig. 4J

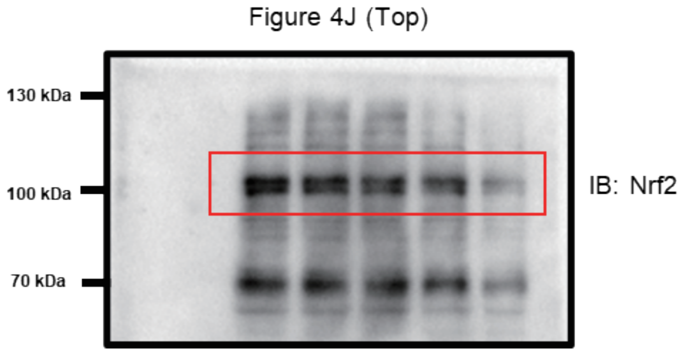

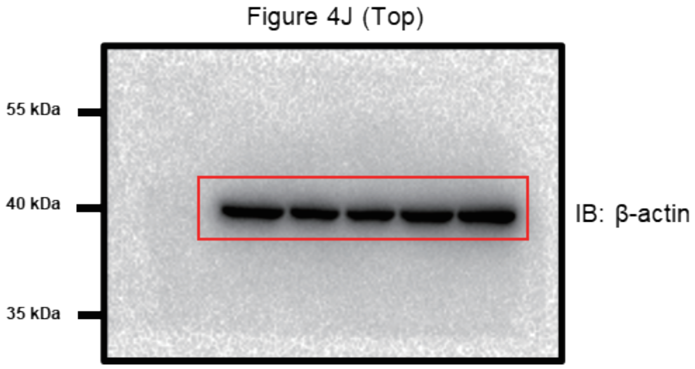

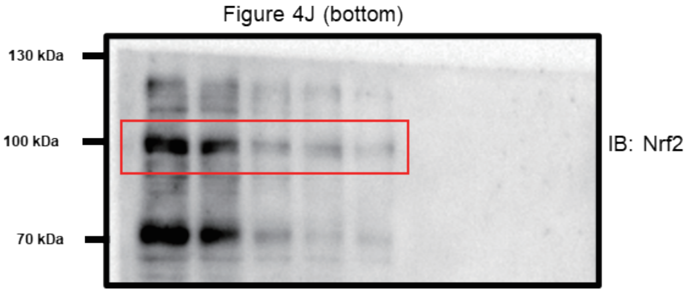

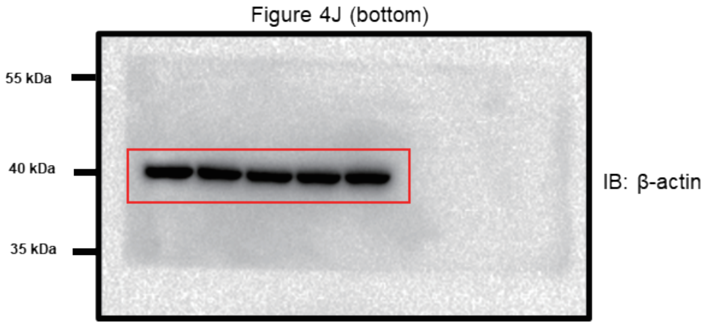

Western Blot: Extended Data Fig. 4K

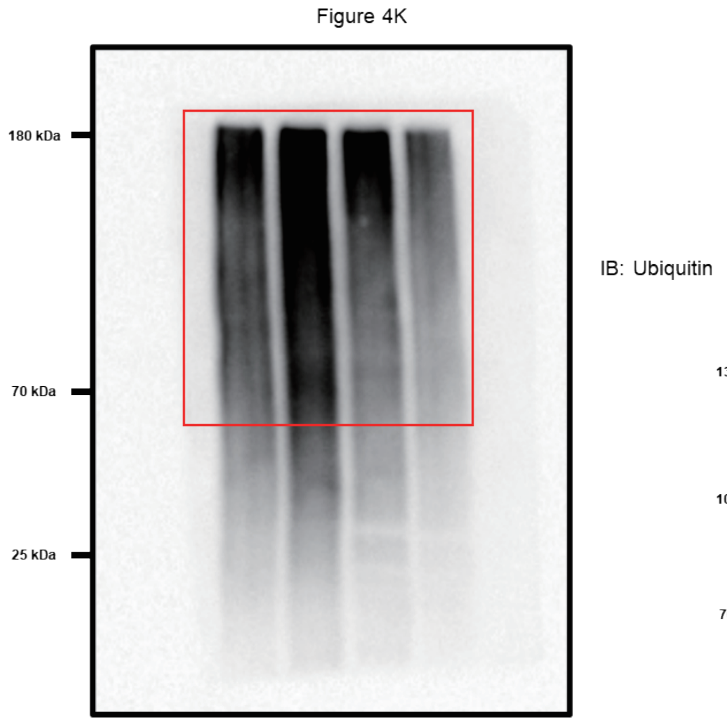

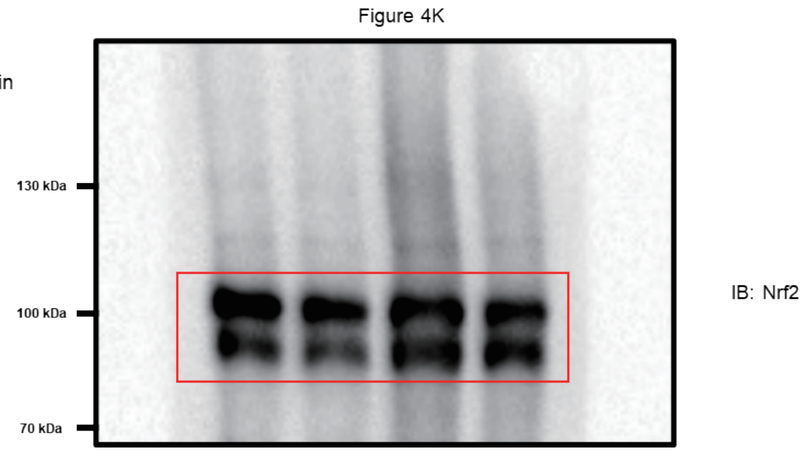

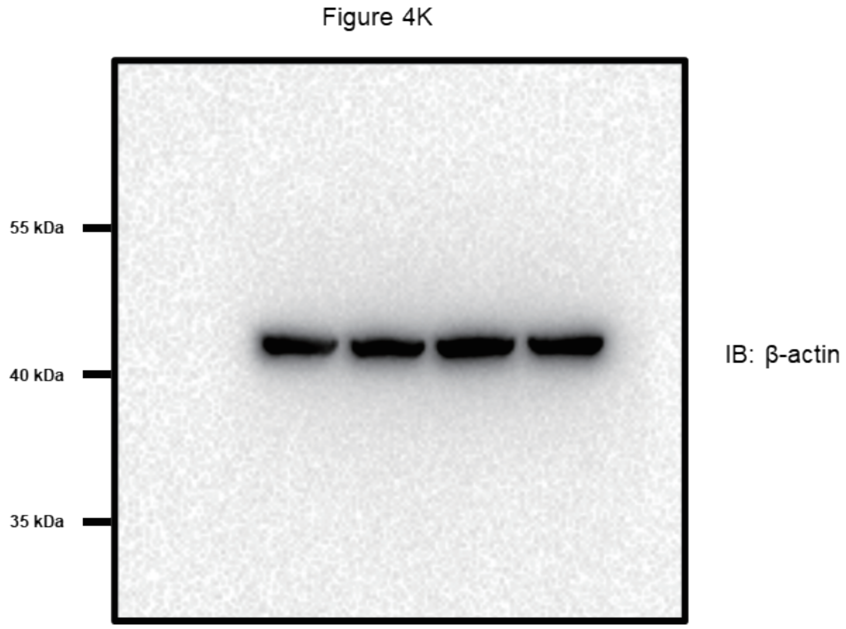

Supplement: Supplementary file 5 — Original Date for Western blots [file 41419_2022_5192_MOESM5_ESM.pdf]
