# Supplementary figures and images for: Long noncoding RNA LINC00239 inhibits ferroptosis in colorectal cancer by binding to Keap1 to stabilize Nrf2
Source: Cell Death Dis. 2022 Aug 29;13(8):742. doi: 10.1038/s41419-022-05192-y (PMC9424287; doi:10.1038/s41419-022-05192-y)

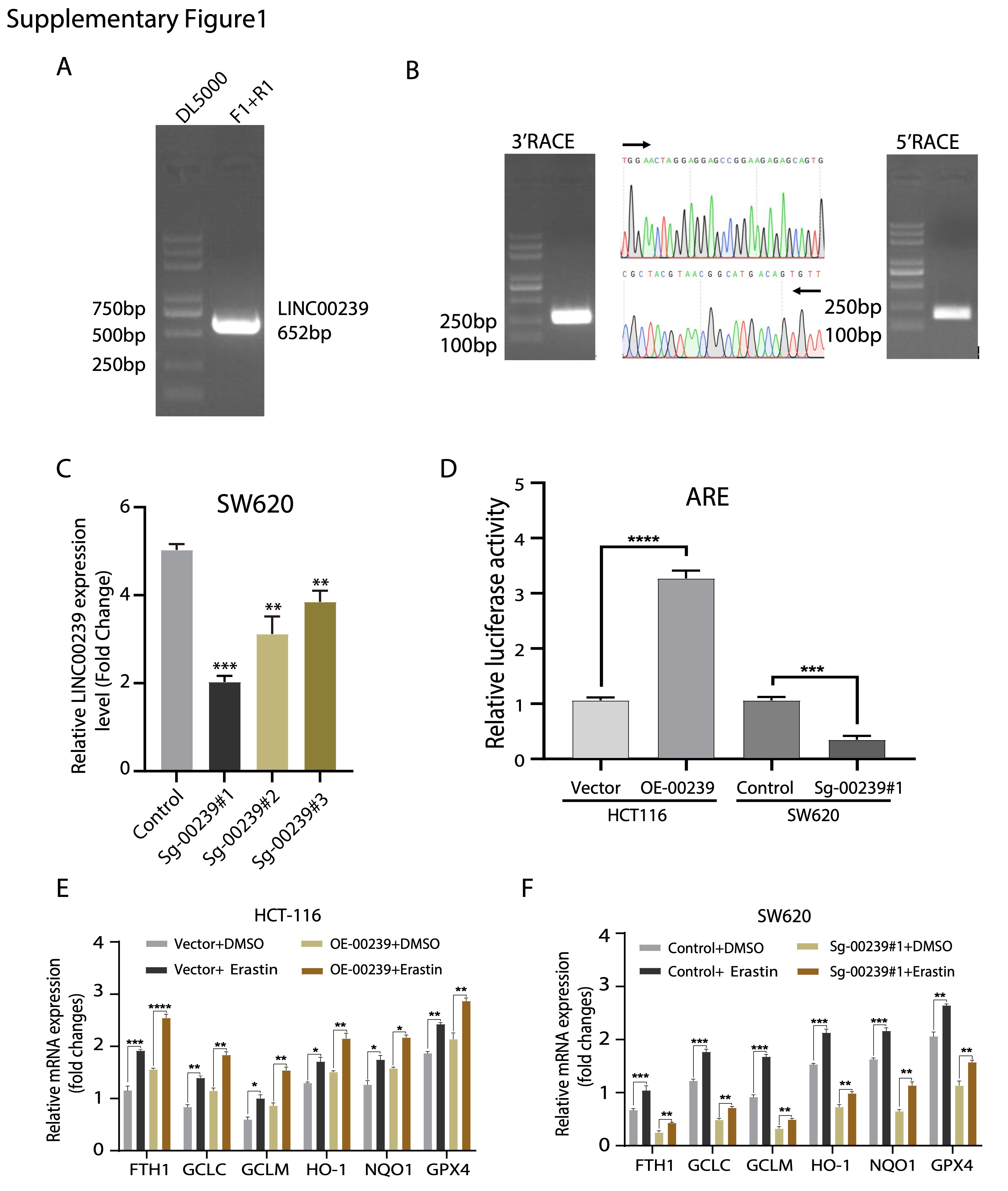

Supplement: Supplementary file 6 — Supplementary Figure 1 [file 41419_2022_5192_MOESM6_ESM.jpg]

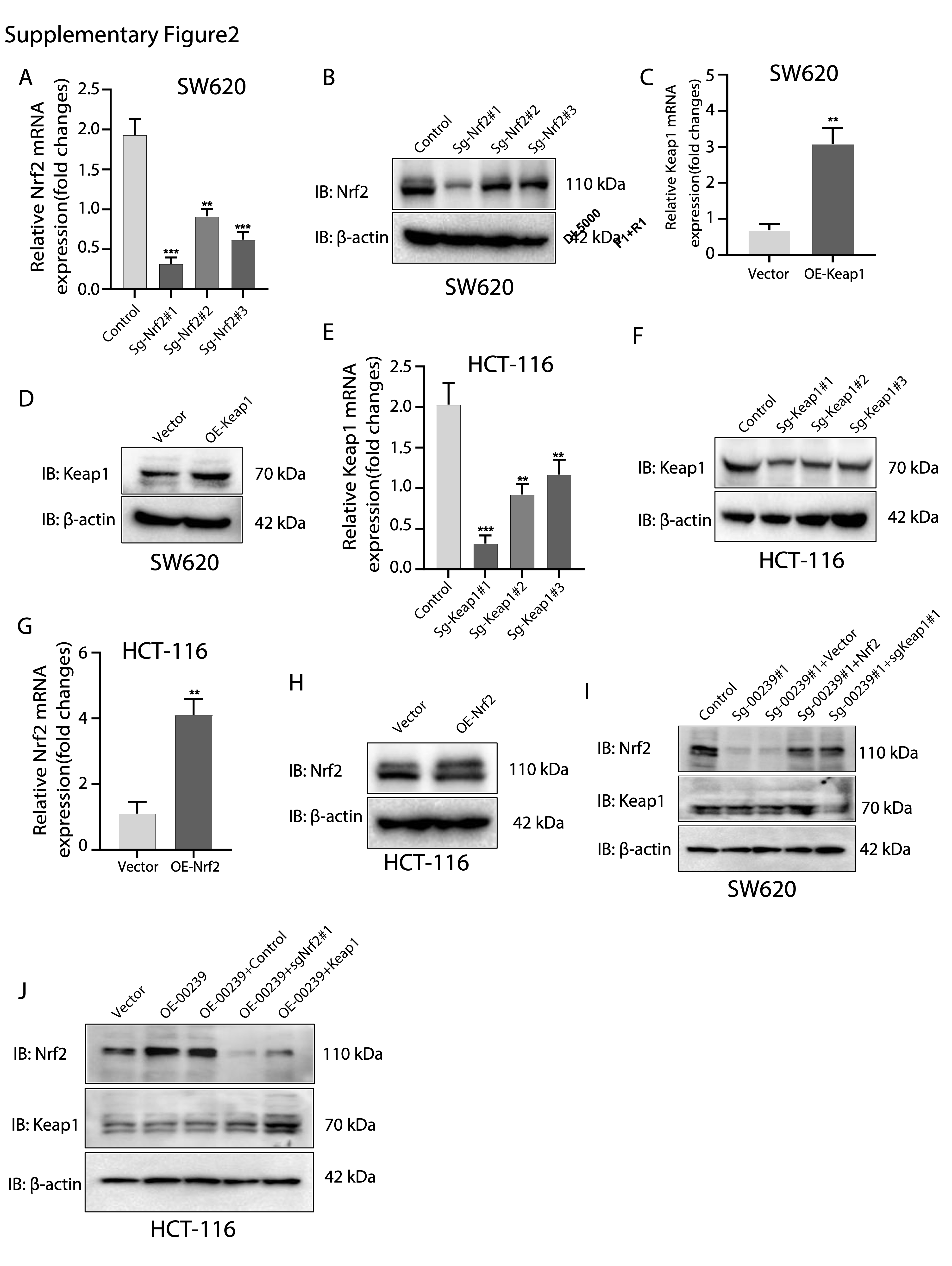

Supplement: Supplementary file 7 — Supplementary Figure 2 [file 41419_2022_5192_MOESM7_ESM.png]

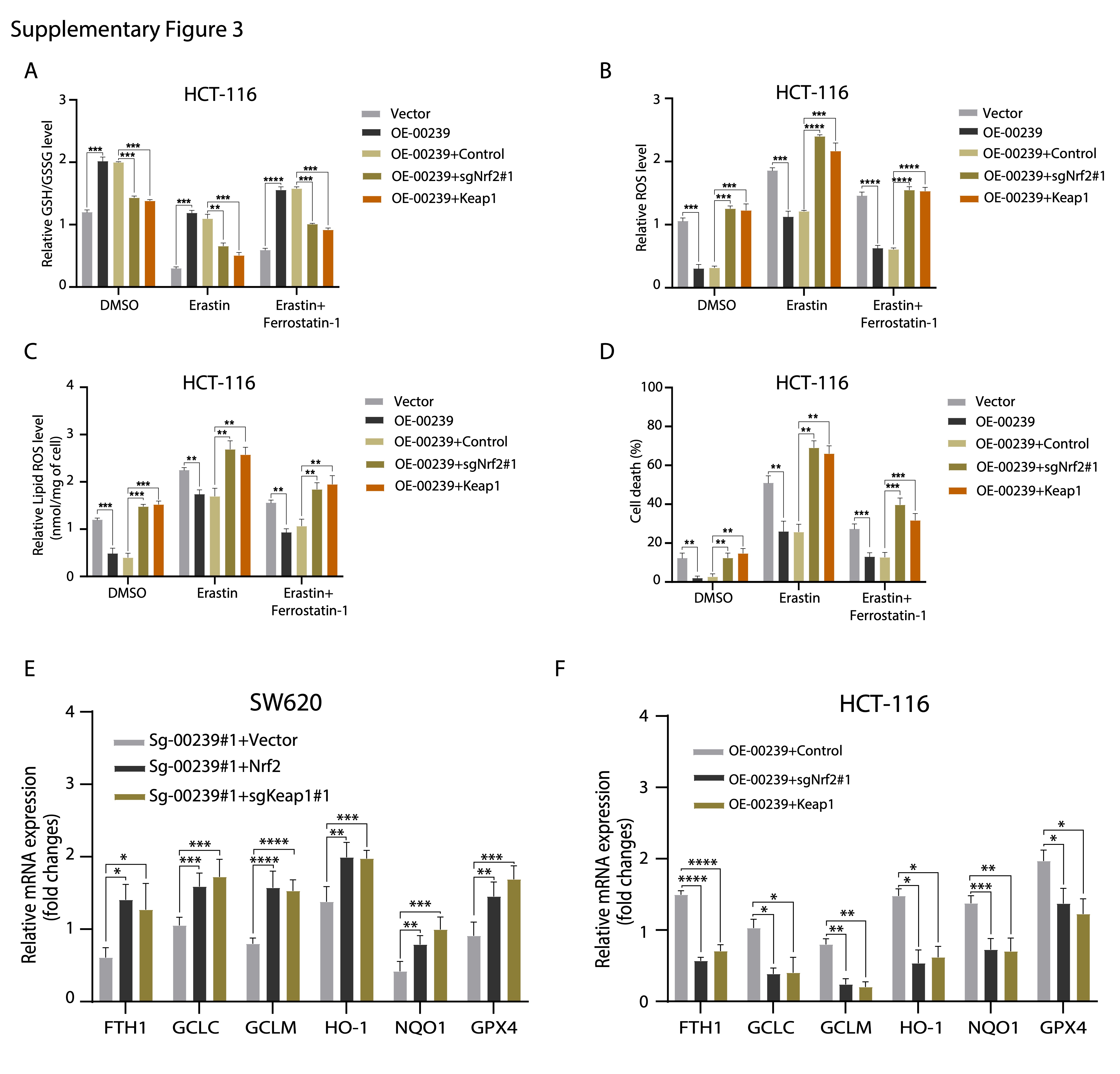

Supplement: Supplementary file 8 — Supplementary Figure 3 [file 41419_2022_5192_MOESM8_ESM.jpg]

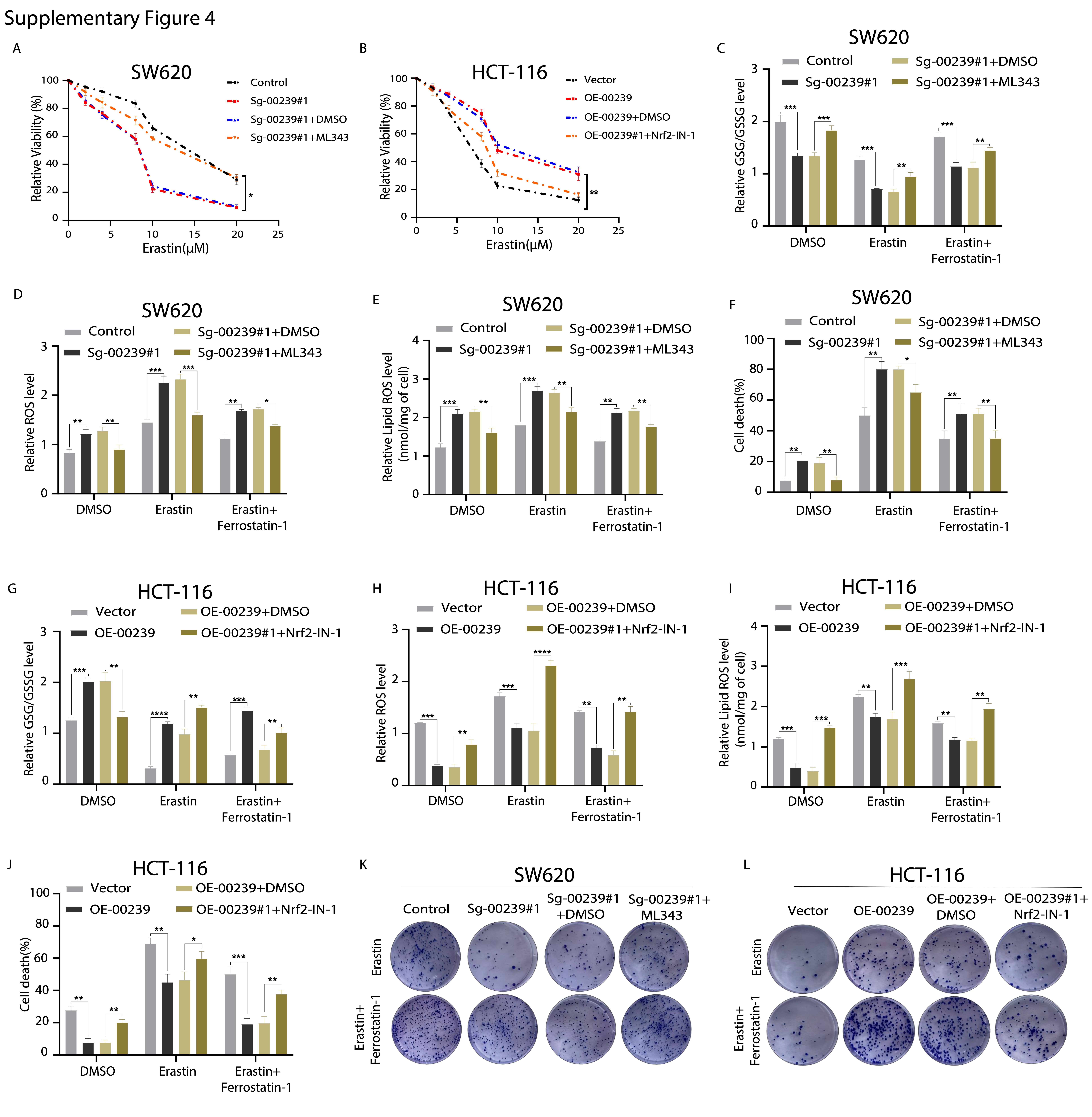

Supplement: Supplementary file 9 — Supplementary Figure 4 [file 41419_2022_5192_MOESM9_ESM.jpg]
